# Confinement promotes nematic alignment of spindle-shaped cells during *Drosophila* embryogenesis

**DOI:** 10.1242/dev.202577

**Published:** 2024-06-28

**Authors:** Tirthankar Ray, Damo Shi, Tony J. C. Harris

**Affiliations:** Department of Cell & Systems Biology, University of Toronto, Toronto, Ontario M5S 3G5, Canada

**Keywords:** Spindle-shape cells, Amnioserosa, Confinement, Nematic order, *Drosophila* embryo

## Abstract

Tissue morphogenesis is often controlled by actomyosin networks pulling on adherens junctions (AJs), but junctional myosin levels vary. At an extreme, the *Drosophila* embryo amnioserosa forms a horseshoe-shaped strip of aligned, spindle-shaped cells lacking junctional myosin. What are the bases of amnioserosal cell interactions and alignment? Compared with surrounding tissue, we find that amnioserosal AJ continuity has lesser dependence on α-catenin, the mediator of AJ-actomyosin association, and greater dependence on Bazooka/Par-3, a junction-associated scaffold protein. Microtubule bundles also run along amnioserosal AJs and support their long-range curvilinearity. Amnioserosal confinement is apparent from partial overlap of its spindle-shaped cells, its outward bulging from surrounding tissue and from compressive stress detected within the amnioserosa. Genetic manipulations that alter amnioserosal confinement by surrounding tissue also result in amnioserosal cells losing alignment and gaining topological defects characteristic of nematically ordered systems. With Bazooka depletion, confinement by surrounding tissue appears to be relatively normal and amnioserosal cells align despite their AJ fragmentation. Overall, the fully elongated amnioserosa appears to form through tissue-autonomous generation of spindle-shaped cells that nematically align in response to confinement by surrounding tissue.

## Teaser

**Summary:** Spindle-shaped amnioserosal cells display distinctive cell-cell adhesion, compressed tissue organization within a narrow strip of the embryo surface and nematic alignment due to confinement by surrounding tissue.

## INTRODUCTION

Epithelial morphogenesis involves tissue-autonomous activities that change cell shapes and interactions (Clarke and Martin, 2021; Fernandez-Gonzalez and Harris, 2023; Paré and Zallen, 2020) as well as physical interactions between neighbouring tissues that coordinate overall embryogenesis (Collinet and Lecuit, 2021; Villedieu et al., 2020). A full understanding of embryo development requires characterizing both the physical dynamics of individual tissues and the physical interplay of interconnected tissues. Cytoskeletal networks are key to driving tissue-autonomous morphogenesis, and differences in cytoskeletal networks between tissues are relevant to tissue-tissue interactions.

Actomyosin network engagement with AJs drives contractile activities within tissues, and contraction of one tissue can also pull neighbouring tissues into new shapes. Within tissues, actomyosin networks pull on the cadherin-catenin complexes of AJs, eliciting α-catenin conformational changes that enhance F-actin binding (Ishiyama et al., 2018), and recruit additional F-actin binding proteins (Charras and Yap, 2018). When these mechanically-induced enhancements to the AJ-actomyosin linkage are lacking, tissue-wide AJ-cytoskeleton networks fragment as they gain myosin-based contractility (Martin et al., 2010; Sawyer et al., 2009; Sheppard et al., 2023). Normal AJ-actomyosin engagements coordinate whole-tissue changes, including invagination by actomyosin-driven apical constriction and convergent-extension by actomyosin-mediated neighbour exchanges (Clarke and Martin, 2021; Fernandez-Gonzalez and Harris, 2023; Paré and Zallen, 2020). Moreover, actomyosin-based re-shaping of one tissue can physically affect attached tissues (Agarwal and Zaidel-Bar, 2021; Bruce and Heisenberg, 2020; Collinet et al., 2015; Gustafson et al., 2022; Lye et al., 2015; Moon and Xiong, 2022). Significantly, junctional actomyosin levels vary greatly across interconnected embryonic tissues, and this variation helps to coordinate their morphogenesis (Rauzi et al., 2015; Streichan et al., 2018). However, tissues with low levels of junctional actomyosin are understudied.

Microtubule (MT) networks are another contributor to AJ positioning and epithelial organization. In addition to transporting vesicular and signalling cargo (Burute and Kapitein, 2019), MTs can exert and withstand pushing forces (Brouhard and Rice, 2018; Kikumoto et al., 2006). For example, MT arrays spanning the apical domains of developing *Drosophila* wing cells counteract the contraction of junctional actomyosin networks to maintain cell shape (Singh et al., 2018). AJ-MT associations also occur in contexts with minimal actomyosin involvement, as exemplified in the *Drosophila* embryo. During blastoderm cellularization, centrosomal MT arrays of each cell gain an apical-basal polarity that directs apicolateral AJ assembly via the scaffold protein Bazooka (Baz/Par-3) (Harris and Peifer, 2005). Global downregulation of this mechanism is part of a shift to actomyosin-based junctional remodelling in the germband (Jiang et al., 2015). However, in contrast to the germband, junctional actomyosin accumulation remains low in neighbouring dorsal tissue (Bertet et al., 2004). Following cellularization of this dorsal tissue, dorsal folds form independently of actomyosin activity and are instead shaped by both basally-directed shifts of Baz-organized AJs (Wang et al., 2012) and MT-based rounding of their apical domains (Takeda et al., 2018). Cells spanning these dorsal folds then reorganize into the fully elongated amnioserosa.

**Fig. 1.**
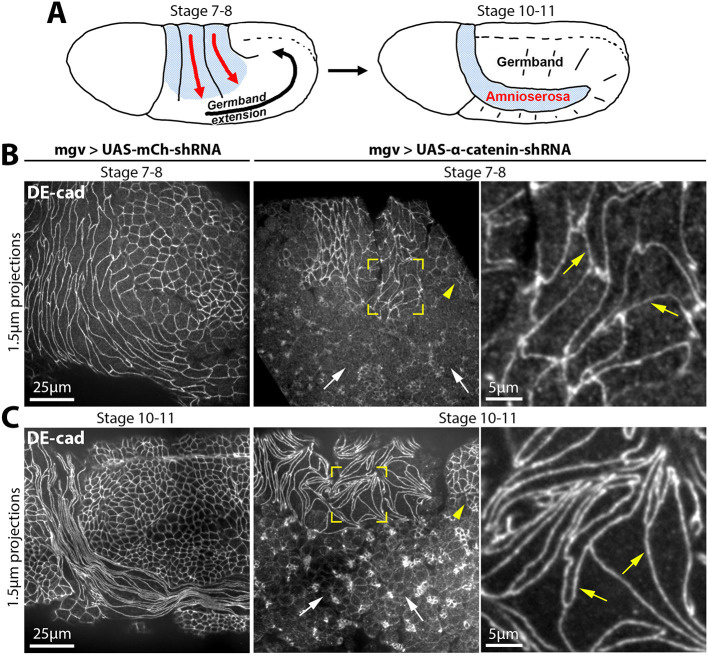
**AJ continuity is less dependent on α-catenin in the amnioserosa than in the germband.** (A) Amnioserosa elongation (red arrows) is coupled with germband extension (black arrow) at stages 7-8. After completion of germband extension, the fully elongated amnioserosa is confined to a horseshoe-shaped strip (blue) surrounded by the germband at stages 10-11 (the horseshoe-shaped strip continues on opposite side of the embryo). Our analyses focused on the lateral side of the embryo, away from the head. (B,C) DE-cad staining of α-catenin RNAi and mCherry RNAi embryos at stages 7-8 (B) and at stages 10-11 (C). mCherry RNAi embryos display continuous DE-cad staining along cell-cell junctions in both the amnioserosa and germband. Seen in 7/7 and 18/18 embryos at stages 7-8 and 10-11, respectively. In α-catenin RNAi embryos, yellow brackets show continuous DE-cad staining along cell-cell junctions throughout the amnioserosa (also see yellow arrows in corresponding magnified views, right). White arrows indicate fragmentation of DE-cad-positive cell-cell contacts throughout the germband. Small patches of dorsal tissue also retained junctional DE-cad (yellow arrowheads). Seen in 6/6 and 19/19 α-catenin 33430 RNAi embryos with disrupted germband at stages 7-8 and 10-11, respectively. Seen in 6/6 α-catenin 38987 RNAi embryos with disrupted germband at stage 10-11.

Amnioserosa morphogenesis is closely coupled with germband extension. As the germband converges ventrally, the attached amnioserosa spreads from the dorsal surface down each side of the embryo. As the germband extends around the posterior pole and over the dorsal surface, the attached amnioserosa fills the intervening space, gaining a thin, horseshoe-like shape (Fig. 1A) (Hartenstein, 1993). Amnioserosa elongation involves tissue-autonomous cell shape changes, with neither cell divisions nor neighbour exchanges (Pope and Harris, 2008). The initially tall, columnar cells rotate their long axes by 90° into the plane of the tissue and extend their apical circumferences into spindle-like shapes. This cell shape change involves MT-based elongation of the apical circumference of each amnioserosal cell. After fully elongating in the plane of the tissue, spindle-shaped amnioserosal cells display strong MT bundles along their lengths and lack junctional myosin. This extensile activity is tissue autonomous. Individual cell elongation is absent in mutants that fail to specify the amnioserosa but does occur in mutants that fail germband extension. However, normal movement of the germband is needed for the overall amnioserosa to gain its horseshoe-like shape and for normal alignment of amnioserosal cells along this shape (Pope and Harris, 2008). Indicating reciprocal effects, amnioserosa elongation is needed for proper germband extension (Pope and Harris, 2008; Rushlow and Levine, 1990), and gaining an array of elongated and aligned amnioserosal cells contributes to subsequent germband retraction (Lynch et al., 2013; McCleery et al., 2019). What are the AJ-cytoskeleton, cell-cell and tissue-tissue interactions that underlie the alignment of spindle-shaped amnioserosal cells?

Cells can align with each other in two main ways. Across a tissue, cell-cell contacts can align through the coordinated planar polarization of molecular complexes (Devenport, 2014), including supracellular actomyosin cables (Fernandez-Gonzalez and Harris, 2023). Alternately, in populations of dynamically interacting rod- or spindle-shaped cells, long axes of cells can align spontaneously and gain nematic order, an effect that also explains alignments of logs in rivers, liquid crystals and reconstituted cytoskeletal networks (Balasubramaniam et al., 2022; Saw et al., 2018). Nematic order is strongly influenced by non-autonomous boundary effects. Of potential relevance to amnioserosal morphogenesis, confinement within long, narrow strips promotes the global alignment of spindle-shaped mammalian cell types in culture (Duclos et al., 2018, 2014). Reduced confinement results in characteristic topological defects within these cellular arrays (Duclos et al., 2014). Such defects also arise with excessive confinement, as shown for spindle-shaped cells (Duclos et al., 2017), and reconstituted extensile MTs (Opathalage et al., 2019). These topological defects are sites where alignment is lost. For example, elongated elements can become arranged in a local aster, referred to as a +1 defect. However, more energetically favourable configurations are comet-like +1/2 defects and threefold symmetric −1/2 defects, which are both signatures of nematically ordered systems (Balasubramaniam et al., 2022; Saw et al., 2018) (see schematics in Fig. 6C). In culture, nematic order has been linked to cell differentiation, sorting and death (Balasubramaniam et al., 2022; Saw et al., 2018). However, nematic order is less studied during animal development. Gain of nematic order can explain actomyosin network alignment in the pseudocleavage furrow of the one-cell *Caenorhabditis elegans* embryo (Reymann et al., 2016) and the arrangement of hepatocytes in the mouse liver (Morales-Navarrete et al., 2019). Topological defects in nematically-ordered ectodermal actin cables have been linked to body plan patterning during Hydra development (Maroudas-Sacks et al., 2021; Wang et al., 2023). We hypothesized that elongating amnioserosal cells gain nematic order when confined by the surrounding germband.

Our data characterize the cell-cell interactions of spindle-shaped amnioserosal cells and show that their alignment correlates with a compressed state, confined by the surrounding germband. Genetic manipulations that alter this confinement result in loss of amnioserosal cell alignment and gain of +1/2 and −1/2 topological defects characteristic of nematically ordered systems. Even with amnioserosal AJ fragmentation, MT arrays of amnioserosal cells display alignment when confined in a narrow strip by the germband. Overall, we conclude that tissue-autonomous development of distinctive cell shapes and cell-cell interactions within the amnioserosa allows its cells to gain nematic alignment in response to non-autonomous confinement by the germband.

## RESULTS

### Amnioserosal AJs rely less on α-catenin than germband AJs

During formation and at full elongation, the amnioserosa has relatively low junctional myosin compared with the germband (Bertet et al., 2004; Pope and Harris, 2008), a difference we confirmed and quantified in the fully elongated tissue at stage 10-11 (Fig. S1A,B). Phalloidin staining also revealed that F-actin was less restricted to AJs in the amnioserosa versus the germband at stage 10-11 (Fig. S1C). Thus, compared with the germband, amnioserosal AJs lack myosin association, and display less focused F-actin association.

To test the relative dependence of the amnioserosa and germband on α-catenin, we expressed an α-catenin short hairpin RNA (shRNA) construct previously shown to disrupt germband cell-cell adhesion (Sheppard et al., 2023), and a distinct α-catenin shRNA construct for confirmation. The shRNAs were expressed maternally to deplete early embryo supplies of α-catenin. Staining for α-catenin showed that its junctional levels were depleted by a similar degree in both the germband and the amnioserosa (Fig. S2A-C). Embryos were stained with DE-cad to assess AJ continuity across cell-cell contacts of the amnioserosa and the germband. At stage 7-8 and stage 10-11, the germband displayed dramatic loss of AJ continuity with α-catenin RNAi (Fig. 1B,C, white arrows show discontinuities), whereas germband AJs were normal in control RNAi embryos. In striking contrast, the junctional distribution of DE-cad was maintained in the amnioserosa of α-catenin RNAi embryos at both stage 7-8 and stage 10-11 (Fig. 1B,C, yellow brackets and arrows), similar to controls. Although junctional DE-cad was maintained in the amnioserosa of α-catenin RNAi embryos, the amnioserosal cells lacked normal alignment with each other (Fig. 1B,C), consistent with abnormal amnioserosal cell arrangements reported for embryos depleted of Armadillo/β-catenin (Pope and Harris, 2008). A patch of dorsal tissue neighbouring the amnioserosa often also maintained AJs with α-catenin RNAi (Fig. 1B,C; yellow arrowheads), but was not characterized further. Overall, these data show that the amnioserosa is less reliant on α-catenin to maintain AJ continuity compared with the germband. This lower reliance on α-catenin could be due to the absence of specific challenges to AJs in the amnioserosa, such as its lack of neighbour exchanges and cell divisions (Pope and Harris, 2008), or to the presence of distinct mechanisms of AJ stabilization, or both.

### Amnioserosal AJs show a uniquely punctate distribution of Baz, which is required for AJ continuity

**Fig. 2.**
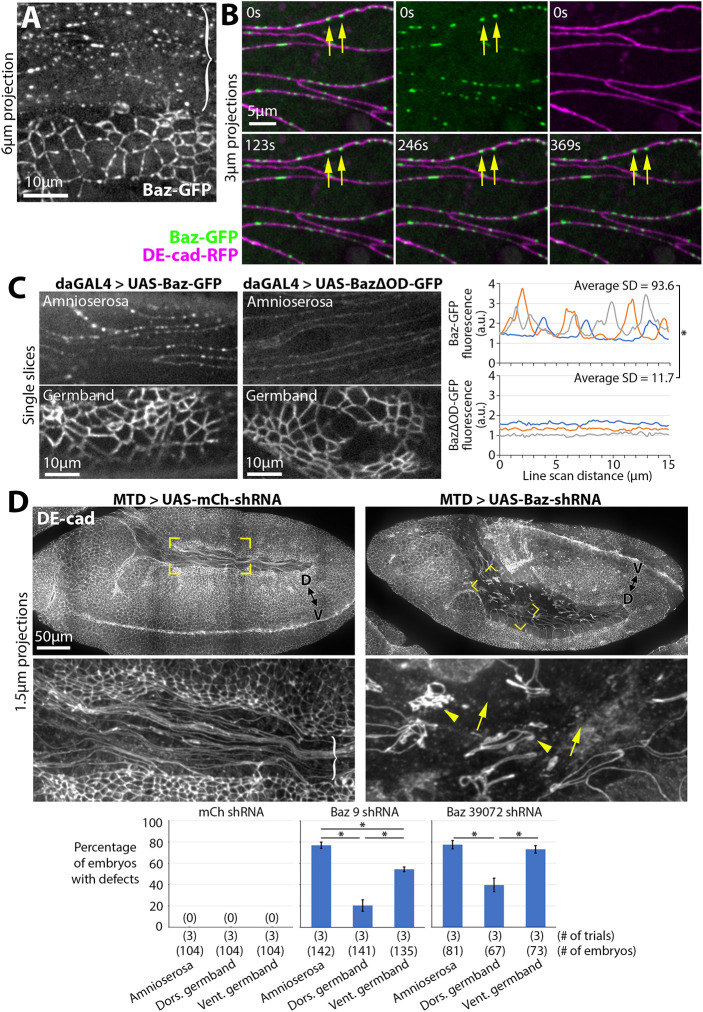
**Bazooka forms distinctive puncta in the amnioserosa and is required for amnioserosal AJ continuity.** (A) Live-imaged stage 10-11 embryo with distinctive Baz-GFP puncta in the amnioserosa (bracket) compared with the germband below. Seen in 12/12 embryos. (B) Dual live-imaging of Baz-GFP and DE-cad-RFP in stage 10-11 amnioserosal cells. Long-lasting Baz-GFP puncta (yellow arrows) exist along cell-cell contacts with relatively even distributions of DE-cad-RFP. Seen in 7/7 embryos. (C) Live-imaged stage 10-11 embryos overexpressing Baz-GFP or BazΔOD-GFP. Baz-GFP displays strong puncta in the amnioserosa (11/11 embryos), whereas BazΔOD-GFP shows a weaker and more diffuse localization along amnioserosal cell-cell contacts (12/12 embryos). Line scans (right) quantify fluorescence intensities along amnioserosal cell-cell contacts of three embryos overexpressing either Baz-GFP or BazΔOD-GFP. Average standard deviation of the Baz construct signal of each set of three embryos shown. Asterisk indicates significant difference between the standard deviations of signal calculated for each set of three embryos (*P*<0.05; Mann–Whitney *U*-test). (D) Projections of stage 10-11 mCherry RNAi and Baz RNAi embryos stained with DE-cad. Yellow brackets indicate magnified amnioserosal regions shown below. In the mCherry RNAi embryo, white bracket indicates amnioserosal region with intact AJs. In the Baz RNAi embryo, yellow arrows indicate fragmentation of amnioserosal AJs and yellow arrowheads indicate abnormal aggregation of amnioserosal AJs. The dorsal-ventral (D-V) axis of the germband is indicated in each case. Graphs show AJ defect frequencies in the amnioserosa, dorsal germband and ventral germband of mCherry RNAi, Baz 9 RNAi and Baz 39072 RNAi embryos at stages 10-11 (mean±s.d. of three trials each). Asterisks indicate significant differences between average frequencies calculated for each of three trials (*P<0.05; Mann–Whitney *U*-test).

Baz is a scaffold protein known to organize AJs in contexts with low junctional myosin (de Matos Simões et al., 2010; Harris and Peifer, 2004, 2005; Wang et al., 2012; Zallen and Wieschaus, 2004). Genetic interaction studies have also implicated Baz in promoting amnioserosa AJ continuity (Pope and Harris, 2008; Shao et al., 2010). To further assess the involvement of Baz in amnioserosa cell-cell adhesion, we probed Baz localization. Live imaging of endogenously expressed Baz-GFP revealed prominent, broadly-spaced puncta in the amnioserosa (Fig. 2A, bracket). In the germband, Baz-GFP displayed a more even distribution, or closely spaced puncta, along AJs (Fig. 2A). To determine the localization and dynamics of the amnioserosa puncta, Baz-GFP was dual-imaged with DE-cad-RFP. Baz puncta were at the level of AJs and maintained their junctional positions for minutes (Fig. 2B, yellow arrows). In contrast to Baz-GFP, DE-cad-RFP displayed a relatively even distribution along amnioserosa AJs (Fig. 2B), suggesting the Baz-GFP puncta are independent of DE-cad clustering or of local accumulations of plasma membrane. The Baz puncta were not maintained with embryo fixation (Fig. S2D), but were detected by live imaging of a distinct, overexpressed Baz-GFP construct (Fig. 2C). A derivative of the overexpressed Baz-GFP construct with a deletion of a known oligomerization domain (Benton and St Johnston, 2003), BazΔOD-GFP, failed to form the amnioserosa puncta (Fig. 2C). Thus, Baz forms unique clusters along AJs of the fully elongated amnioserosa via homo-oligomerization.

To test whether the puncta are unique to Baz, we probed the localization of known binding partners of Baz, aPKC and Par-6 (Ohno, 2001). Live imaging of endogenously expressed aPKC-GFP and Par-6-GFP at stage 10-11 showed that they both formed large, broadly-spaced puncta along amnioserosa cell-cell contacts (Fig. S3A,B, white brackets), in contrast to more even distributions along germband cell-cell contacts (Fig. S3A,B). The sensitivity of the puncta to fixation prevented colocalization tests of endogenously expressed proteins by immunostaining. Nonetheless, these data suggest that Baz forms clusters with its known partners aPKC and Par-6 along amnioserosa AJs.

To test whether Baz affects amnioserosal AJs, we depleted embryos of Baz by maternal expression of two distinct shRNA constructs. Staining for Baz showed that its junctional levels were depleted by a similar degree in both the germband and the amnioserosa (Fig. S2D-F). We stained for DE-cad to assess AJ continuity. In contrast to the continuous amnioserosal AJs of control RNAi embryos (Fig. 2D, white bracket), Baz RNAi embryos showed large amnioserosal regions with AJ loss (Fig. 2D, yellow arrows) close to regions with abnormal AJ coalescence into ring-shaped structures (Fig. 2D; yellow arrowheads). The neighbouring dorsal germband was affected less frequently in Baz RNAi embryos (Fig. 2D), whereas junction fragmentation was frequent in the ventral germband (Fig. 2D), consistent with a known role of Baz in maintaining AJs in the ventral neurectoderm (Harris and Tepass, 2008). AJ fragmentation was a relatively specific effect of Baz RNAi on amnioserosal cells, as stainings of nuclei (Fig. S4), the basolateral membrane marker Discs large (Dlg) (Fig. S4) and MTs (Fig. 7A) appeared to be unaffected compared with control RNAi embryos. Thus, the amnioserosa is a tissue type with greater dependence on Baz for AJ continuity.

### Strong MT bundles run along amnioserosal AJs and are required for AJ curvilinearity

**Fig. 3.**
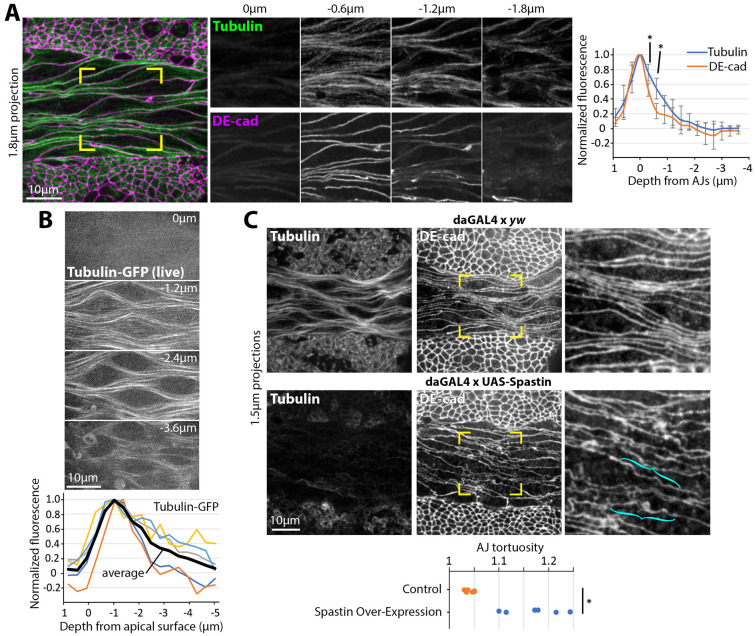
**MTs form strong bundles along amnioserosal AJs and are required for AJ curvilinearity.** (A) Stage 10-11 *yw* embryo stained for tubulin and DE-cad. Yellow brackets indicate amnioserosal region shown in *z*-sections to right, where 0 µm is apical and the strongest signal of both MTs and AJs is at −0.6 µm. Graph shows tubulin and DE-cad levels from apical to basal sections of amnioserosa cells. Signal intensities normalized to section with maximum intensity and shown as mean±s.d., and 0 µm set as the section with maximum DE-cad levels. Five co-stained embryos quantified. Asterisks indicate significantly higher normalized tubulin levels versus normalized DE-cad levels at sections below that with maximum DE-cad levels (*P<0.05; Mann–Whitney *U*-test). (B) Apical to basal sections of amnioserosal cells of a live stage 10-11 tubulin-GFP embryo. Graph shows peaks of normalized tubulin-GFP fluorescence in sections just below the apical surface. Five embryos were quantified and a line (black) with average normalized intensities indicated. (C) Projections of tubulin and DE-cad staining of a stage 10-11 Spastin-overexpressing embryo compared with control. MT networks are diminished by Spastin overexpression. Yellow brackets indicate magnified amnioserosal regions to right. Cyan brackets indicate abnormal local undulations of AJs in response to Spastin overexpression. Seen for 12/12 control embryos and for 13/13 Spastin-overexpression embryos with diminished MT networks. Graph quantifies a significant increase in AJ tortuosity in Spastin-overexpression embryos with diminished MT networks compared with control embryos (*P<0.001; *N*=6 embryos each; Mann–Whitney *U*-test).

Cell-cell junctions of fully elongated amnioserosal cells are curvilinear (displaying interconnected curved and straight segments). Although actomyosin-based tensile stress can straighten AJs along their length (Lenne et al., 2021), the lack of junctional myosin in the fully elongated amnioserosa prompted us to consider an alternate mechanism that could affect long-range junctional shape. As MT bundles run along the long axis of each amnioserosal cell (Pope and Harris, 2008), we hypothesized that they support the curvilinearity of AJ-based cell-cell contacts in the amnioserosa. To test the degree of association between MT bundles and AJs, we compared their localization in stage 10-11 embryos. Tubulin staining revealed MT bundles running along the length of AJ-based cell-cell contacts in the amnioserosa, with the strongest accumulation of MT bundles at and immediately below AJs in the apicobasal axis (Fig. 3A). A similar distribution of MT bundles was detected by live imaging of Tubulin-GFP (Fig. 3B). Bathing embryos in colchicine, an MT inhibitor, strongly disrupts cell arrangement of the fully elongated amnioserosa (Pope and Harris, 2008). As an alternate approach, we overexpressed the MT-severing enzyme Spastin (Sherwood et al., 2004). In Spastin-overexpressing embryos, the general arrangement of amnioserosal cells was maintained, allowing more specific assessment of effects on AJ curvilinearity. With this approach, depletion of MT networks correlated with increased local tortuosity along DE-cad-based cell-cell contacts (Fig. 3C, cyan brackets). Thus, MT bundles run along amnioserosal AJs and support their long-range curvilinearity.

### The amnioserosa is composed of partially overlapping, spindle-shaped cells

**Fig. 4.**
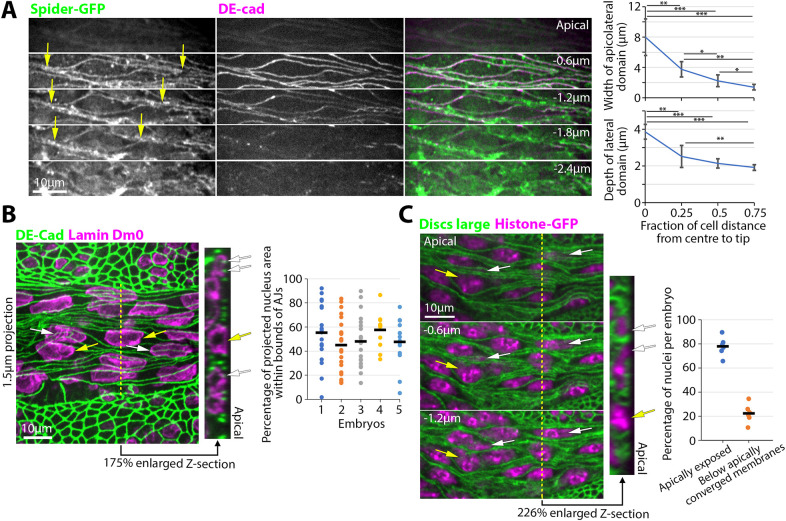
**Amnioserosal cells partially overlap each other.** (A) Apical to basal sections of a stage 10-11 Spider-GFP embryo stained with DE-cad. Yellow arrows indicate shortening of cell length at deeper sections. Graphs quantify tapering of amnioserosa cells at the amnioserosa surface (level of AJs) and in depth. Mean±s.d. shown for five measurements across five embryos (*P<0.05; **P<0.01; ***P<0.001; Mann–Whitney *U*-test). (B) Stage 10-11 *yw* embryo stained for Lamin Dm0 and DE-cad. Surface view and enlarged *z*-section shown (dotted yellow line shows length and position of *z*-section). White arrows indicate nuclei extending beyond AJs of the cell. Yellow arrows indicate nuclei mainly within the bounds of the AJs of the cell. Graph shows percentages of projected nuclear areas extending beyond AJs for five embryos (dots are individual nuclei; bars are means). (C) Single sections from apical (0 µm) toward basal position of a stage 10-11 Histone-GFP embryo stained for Dlg. Dotted yellow line shows length and position of enlarged *z*-section. Yellow arrows show amnioserosal nuclei exposed to the apical surface. White arrows indicate amnioserosal nuclei below apically converging lateral membranes. Graph shows the proportion of amnioserosal nuclei exposed to the apical surface or below apically converging membranes for six embryos (dots are individual embryos, bars are means).

The curvilinearity of amnioserosal AJs is linked to spindle-like amnioserosal cell shapes in surface views. We next investigated the 3D arrangement of cells within the fully elongated amnioserosa, examining whether the spindle-shaped cells are arranged with the same cell perimeters from apical to basal, like floor tiles, or whether the cells partially overlap with each other in 3D. To test the 3D arrangement of spindle-shaped amnioserosal cells, we first fixed stage 10-11 embryos expressing Spider-GFP to detect plasma membranes and stained with DE-cad to mark AJs. At the level of apicolateral AJs, Spider-GFP outlined spindle-like cell shapes, with a bulge in the middle and thin extensions at each end (Fig. 4A). Moving basally, the extensions at each end shortened (Fig. 4A, arrows). Quantifications showed that the extended ends of spindle-shaped amnioserosal cells taper in both the embryo surface plane (*xy*) and in depth (*z*) (Fig. 4A). Thus, spindle-shaped amnioserosal cells do not have the same perimeter from apical to basal. To test whether the extended ends of amnioserosal cells partially overlap with cellular contents of neighbouring amnioserosal cells, we co-stained for DE-cad and Lamin Dm0, a nuclear marker. Consistent with spindle-shapes, at the middle of many cells, the apical circumference widened and was occupied by a nucleus within the bounds of the AJs of one cell (Fig. 4B, yellow arrows). In contrast, nearby cells displayed a narrower middle region and positioning of the nucleus beyond the AJs of one cell and below the contents of neighbouring cells (Fig. 4B; white arrows). Quantifications revealed broad ranges of this overlap across cells within individual embryos (Fig. 4B). To test whether the overlap was due to full stratification of the amnioserosa into distinct cell layers, we stained embryos expressing the nuclear marker Histone-GFP with the plasma membrane marker Dlg. Nuclei were either exposed to the apical surface (Fig. 4C, yellow arrows) or below apically converging plasma membranes (Fig. 4C, white arrows), arguing against full stratification of the cells. Overall, these data indicate that cells of the fully elongated amnioserosa partially overlap with each other.

### The amnioserosa is under compressive stress

**Fig. 5.**
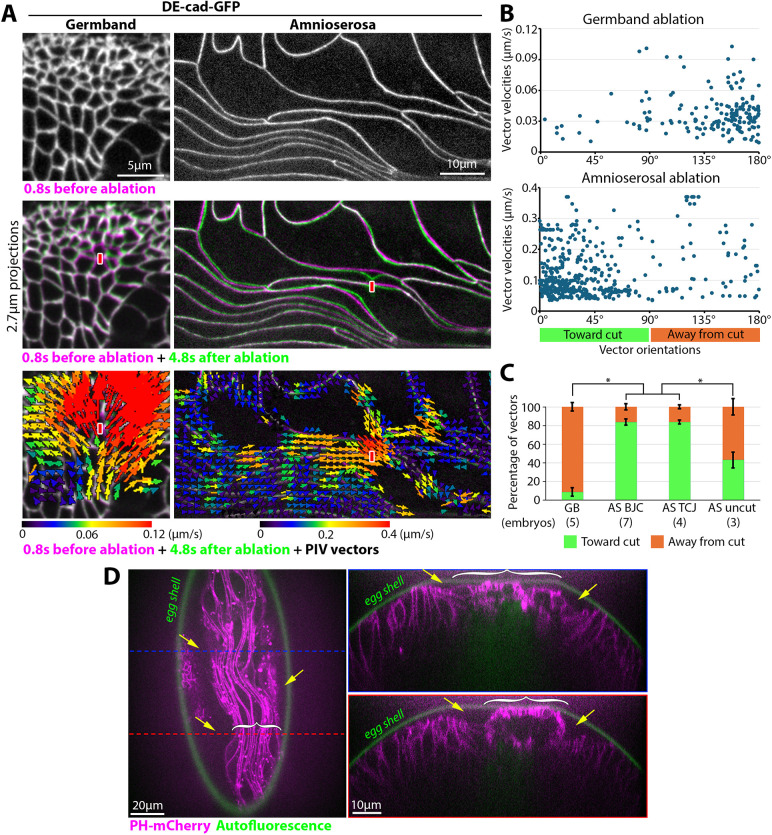
**The amnioserosa is under compressive stress.** (A) Live stage 10-11 DE-cad-GFP embryos before (purple) and after (green) laser ablations of AJs in either the germband or the amnioserosa. Red bars indicate sites of ablation. Tissue movement was away from the ablation site in the germband (seen in seven embryos; Movie 1) and toward the ablation site in the amnioserosa (seen in 17 embryos to various degrees; Movies 2 and 3). At the bottom, the images are overlaid with PIV vectors (colour-coded scales of velocities shown for each tissue). (B) Plots of PIV vector orientations versus PIV vector velocities for the two embryos shown in A. Orientations of less than 90° are toward the cut, and those greater than 90° are away from the cut. (C) Percentages of PIV vectors orientated toward or away from the cut for the dorsal germband (GB), amnioserosal bi-cellular junctions (AS BCJ) and amnioserosal tri-cellular junctions (AS TCJ). PIV vector orientations relative to the centre of the field of view of uncut amnioserosal samples also shown (the velocities of these vectors were low; Fig. S6A). Mean±s.d. shown. Asterisks indicate significant differences for embryo numbers shown (*P<0.05; Mann–Whitney *U*-test). (D) Live stage 10-11 PH-mCherry embryo with plasma membrane in magenta and autofluorescent egg shell in green. Surface view and two cross sections (blue and red lines) shown. Brackets indicate width of amnioserosa. Yellow arrows indicate inward bending of germband on either side of amnioserosa. Between the inward bends, the amnioserosa contacts the egg shell. Seen in 11 embryos.

In pseudostratified tissues, partial overlap of epithelial cells allows them to pack into a smaller space (Lemke and Nelson, 2021). The partial cell overlap of the fully elongated amnioserosa could be organized solely by interactions among amnioserosal cells, or could be promoted by compression from the neighbouring germband. To probe for compressive stress at amnioserosal AJs, we used laser ablation to cut individual AJs in DE-cad-GFP embryos and quantified responses with particle image velocimetry (PIV) analyses (Fig. S5A,B; Materials and Methods). Locally cutting a cell-cell contact of the dorsal germband resulted in recoil of most surrounding junctions away from the cut site (Fig. 5A-C; Movie 1), indicating tensile stress, as expected for a tissue with junctional actomyosin networks (Zulueta-Coarasa and Fernandez-Gonzalez, 2017). In contrast, locally cutting a cell-cell contact of the amnioserosa resulted in displacements of surrounding junctions toward the cut site (Fig. 5A-C; Movies 2 and 3), indicating compressive stress. Similar responses occurred following cuts of curved bi-cellular junctions, straight bi-cellular junctions or tri-cellular junctions (Fig. 5B,C; Fig. S6A), suggesting general compression across the tissue. Displacements were not observed in uncut amnioserosal tissue (Fig. 5C; Fig. S6A; Movie 4). Occasionally, junctions surrounding the cut site moved away, but performing the experiment in embryos co-expressing Histone-RFP and DE-cad-GFP revealed that this effect correlated with cutting of the nucleus below (Fig. S6B). As the inward junctional displacements occurred over long distances relative to the small cut site, we also investigated the effect of the local ablation on the MT bundles that run along the full lengths of amnioserosal cells. In embryos expressing Jupiter-GFP, a marker of MTs, local ablation at a cell-cell contact resulted in apparent MT depolymerization away from the cut site within seconds (Fig. S7A, bracket; quantified in Fig. S7B), and loss of MTs throughout the entirety of the ablated cells within a minute (Fig. S7C). Within seconds, intact MT bundles of surrounding cells also shifted position toward the cut site (Fig. S7A, white arrows). Together, these data indicate that the fully elongated amnioserosa is under compressive stress, possibly withstood by MT bundles running along cell-cell junctions.

Scanning electron micrographs have shown a bulging of the amnioserosa from the embryo surface (Turner and Mahowald, 1977), leading us to hypothesize that the amnioserosa is being compressed by the surrounding germband. To test whether the amnioserosa bulges in live embryos, we imaged a plasma membrane probe, PH-mCherry, at stage 10-11, as well as autofluorescence of the egg shell. In both embryo surface views and embryo cross sections, the neighbouring germband was indented along both sides of the amnioserosa (Fig. 5D). At the indentations, gaps were present between the germband and the outer egg shell. In between the indentations, the amnioserosa closely associated with the egg shell. These observations suggest that the amnioserosa is under compressive stress due to confinement from the germband on either side and from the egg shell above.

### Amnioserosal cell alignment is affected by neighbouring tissue

**Fig. 6.**
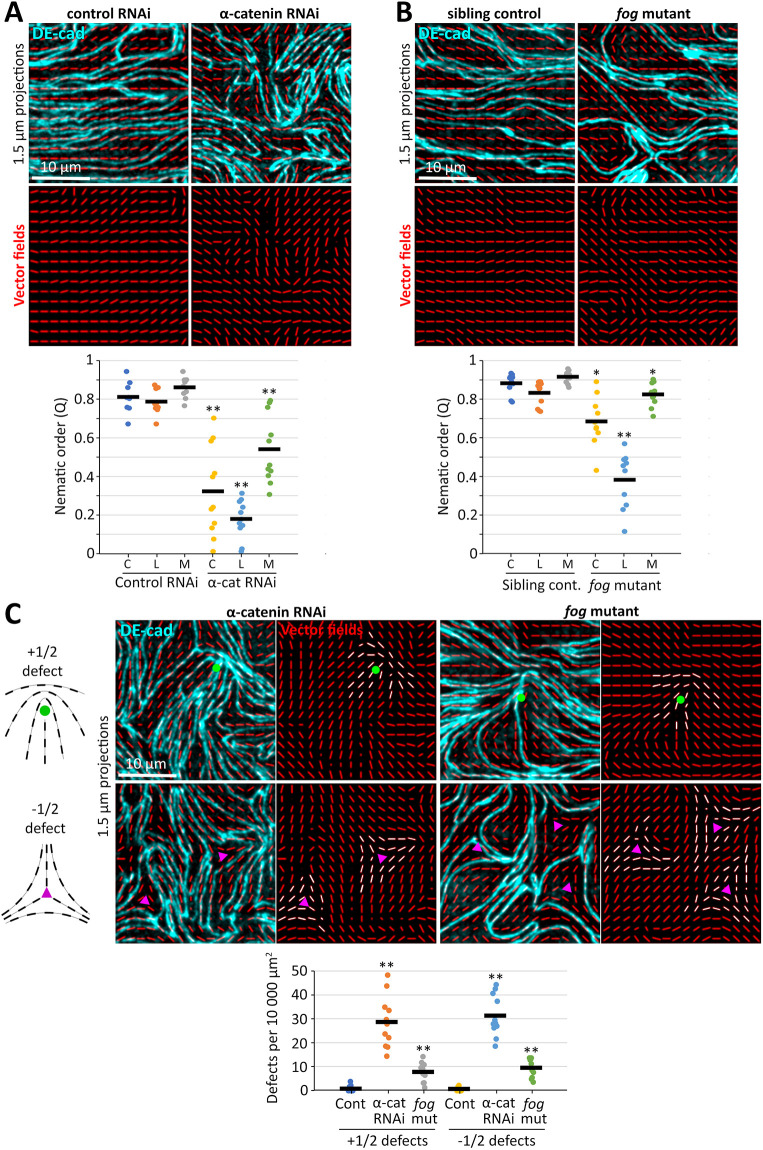
**Loss of amnioserosal cell alignment linked to distinct disruptions of the surrounding germband.** (A) DE-cad staining (cyan) of amnioserosal cells of stage 10-11 mCherry RNAi and α-catenin RNAi embryos overlaid with corresponding vector fields (red). Graph shows nematic order quantifications from the centre region of the amnioserosa (C), the amnioserosa region with the least alignment by eye (L) and the amnioserosa region with the most alignment by eye (M). Each dot represents one embryo; bars show means. α-Catenin RNAi embryos displayed significantly lower amnioserosal alignment for all three regions (**P<0.001; Mann–Whitney *U*-test; nine mCherry RNAi and 11 α-catenin RNAi embryos quantified). (B) DE-cad staining (cyan) of amnioserosal cells of stage 10-11 sibling control and *fog* mutant embryos overlaid with corresponding vector fields (red). Graph shows nematic order quantifications, as in A. *fog* mutants displayed significantly lower amnioserosal alignment for all three regions (*P<0.01; **P<0.001; Mann–Whitney *U*-test; ten sibling controls and 11 *fog* mutants quantified). (C) Schematics show vector arrangements of +1/2 and −1/2 defects. In vector fields based on DE-cad amnioserosal junctional staining of both α-catenin RNAi and *fog* mutant embryos, +1/2 and −1/2 defects indicated with green circles and purple triangles, respectively, and vectors comprising defects indicated in white. Graph shows significantly higher defect frequencies in α-catenin RNAi (11 embryos) and *fog* mutant embryos (10 embryos) versus sibling controls of the *fog* mutants (11 embryos) (**P<0.001; Mann–Whitney *U*-test).

Spindle-shaped mammalian cells gain nematic order when grown to confluence in a narrow strip (Duclos et al., 2014). As amnioserosal cells are confined in a narrow strip, spindle-shaped and aligned, we hypothesized their alignment is based on nematic ordering in response to confinement from the germband. Nematically ordered systems display general alignment of their elongated elements, as well as characteristic topological defects: most commonly comet-like +1/2 defects and threefold symmetric −1/2 defects (Balasubramaniam et al., 2022; Saw et al., 2018) (see schematics in Fig. 6C). To calculate nematic order (Q), we adapted the approach of Duclos et al. (2014). Specifically, images of amnioserosal cells were processed to show alignments of AJs as vector fields, from which nematic order was calculated (Fig. S5C,D; Materials and Methods). To test the effect of germband positioning on amnioserosal cell alignment, we first compared the nematic order of DE-cad-positive cell-cell contacts in control and α-catenin RNAi embryos at stage 10-11. α-Catenin RNAi embryos fail convergent extension of the germband owing to its AJ fragmentation (Sheppard et al., 2023) (Fig. 1B,C). In the amnioserosa, DE-cad-positive cell-cell contacts had substantially higher alignment in control RNAi embryos versus α-catenin RNAi embryos (Fig. 6A). Compared with controls, α-catenin RNAi embryos also had significantly greater frequencies of amnioserosal +1/2 defects and −1/2 defects (Fig. 6C), which were counted manually from vector field images. Live imaging of DE-cad-GFP in α-catenin RNAi embryos revealed that amnioserosal misalignment arose during stages 7-8 as the amnioserosa elongated against an immobile germband and its cells became locally twisted, in contrast to the aligned elongation of amnioserosal cells in controls (Fig. S8A).

Although α-catenin RNAi embryos form continuous AJs in the amnioserosa (Fig. 1B,C), their amnioserosal cell misalignment might arise from defective amnioserosal cell-cell interactions rather than abnormal germband positioning. To test the effect of germband positioning with a perturbation not expected to affect the amnioserosa autonomously, we analyzed *folded gastrulation* (*fog*) mutants. In *fog* mutants, the germband extends improperly during stages 7-8 due to the obstruction of a failed posterior midgut invagination (Sweeton et al., 1991), and *fog* expression is undetectable in the forming amnioserosa (Costa et al., 1994). In stage 10-11 *fog* mutants, we observed excessively expanded areas of amnioserosa compared with controls and, in these regions, less overlap occurred between amnioserosal cells (Fig. S8B). Compared with the amnioserosa of control stage 10-11 embryos, expanded areas of amnioserosa in *fog* mutants displayed less nematic order (Fig. 6B) and more frequent topological defects (Fig. 6C). Together, these data indicate that the amnioserosa becomes misaligned and accumulates topological defects when its expansion is blocked by an immobile germband in α-catenin RNAi embryos, or when its expansion is excessive due to a distorted germband in *fog* mutant embryos. Thus, the amnioserosa displays properties of a nematically ordered system, and its alignment appears to be promoted by confinement from the surrounding germband.

### Amnioserosal cell alignment can occur independently of intact amnioserosal AJs

**Fig. 7.**
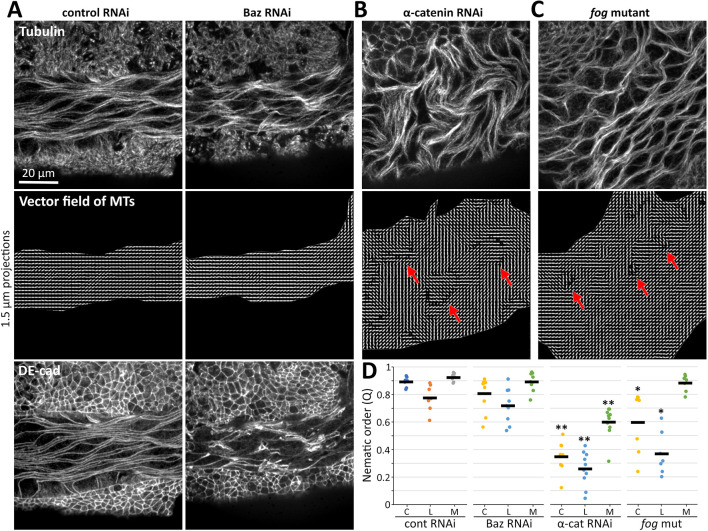
**Amnioserosal cell alignment has independence from amnioserosal AJ continuity.** (A) Stage 10-11 mCherry RNAi and Baz RNAi embryos stained for tubulin and DE-cad. Vector fields of amnioserosal regions based on MT distributions. Baz RNAi embryos with substantial loss of amnioserosal AJs were selected based on DE-cad staining, but their MT-based vector fields were similar to controls. (B,C) Stage 10-11 α-catenin RNAi embryos and stage 10-11 *fog* mutants stained with tubulin. Vector fields based on MT distributions displayed misalignments (red arrows). (D) Nematic order quantifications based on MT distributions in three regions of the amnioserosa, as described in Fig. 6A. Each dot represents one embryo; bars show means. Amnioserosa MT alignment in Baz RNAi embryos (nine embryos) was indistinguishable from control RNAi embryos (seven embryos). Amnioserosa MT alignment was significantly lower in α-catenin RNAi embryos (nine embryos) and *fog* mutants (seven embryos) versus the control RNAi embryos (*P<0.01; **P<0.001; Mann–Whitney *U*-test).

To further probe the basis of amnioserosal cell alignment, we tested the role of intact AJs within the amnioserosa. Specifically, we measured amnioserosal cell alignment in Baz RNAi embryos, in which the amnioserosa loses AJ continuity but remains confined by an intact, surrounding germband (Fig. 2D). We analyzed the alignment of MT bundles in amnioserosal regions with AJ breakdown in stage 10-11 Baz RNAi embryos. We found that nematic order of amnioserosal MT bundles was indistinguishable between control and Baz RNAi embryos (Fig. 7A,D), whereas MT bundle misalignment occurred in α-catenin RNAi and *fog* mutant embryos (Fig. 7B-D). These data suggest that amnioserosal AJ continuity is not required for maintaining nematic order of spindle-shaped amnioserosal cells confined by the surrounding germband.

## DISCUSSION

Our multiscale analysis of the fully elongated amnioserosa identifies organizational properties distinct from those of epithelia with junctional actomyosin networks. Baz is required for amnioserosal AJ continuity, whereas α-catenin is more crucial for germband AJ continuity. MT bundles run along amnioserosal cell-cell contacts and promote their curvilinearity. The fully elongated amnioserosa exists under compressive stress, and its spindle-shaped cells are packed with partial overlap. Confinement of the amnioserosa by the surrounding germband appears to promote nematic alignment of its spindle-shaped cells.

AJ continuity relies on distinct mechanisms in the germband and amnioserosa. Germband extension involves AJ-actomyosin network engagement. Such engagement can induce α-catenin conformational changes that enhance F-actin binding (Ishiyama et al., 2018) and recruit additional mediators of AJ-actin interactions (Charras and Yap, 2018). In embryos depleted of α-catenin, or its reinforcing partner Canoe, actomyosin contraction tears junctional networks apart (Martin et al., 2010; Sawyer et al., 2009), and α-catenin mechanical signal transduction is required to prevent this tearing (Sheppard et al., 2023). In contrast, amnioserosa elongation begins with relatively low junctional myosin (Bertet et al., 2004), which is lost with elongation (Pope and Harris, 2008; Fig. S1A,B). The fully elongated amnioserosa also displays distinctive junctional F-actin (Fig. S1C). More strikingly, α-catenin depletion that fragments germband AJs has no effect on amnioserosal AJ continuity (Fig. 1B,C), which is instead dependent on the scaffold protein Baz (Fig. 2D). Intriguingly, Baz affects AJs in other contexts with low junctional myosin, including *Drosophila* embryo cellularization (Harris and Peifer, 2004) and dorsal fold morphogenesis (Wang et al., 2012), and Baz localizes to myosin-depleted AJs in epithelia with planar polarized junctional myosin (e.g. in the germband; Zallen and Wieschaus, 2004). Along amnioserosal AJs, we found Baz in distinctively large, broadly-spaced and immobile puncta (Fig. 2A,B). The puncta depend on the Baz oligomerization domain (Fig. 2C) and resemble those detected for Baz interaction partners Par-6 and aPKC (Fig. S3). Corresponding intra- and intermolecular interactions of the Baz ortholog Par-3 have been implicated in phase separation (Liu et al., 2020). However, the role of the Baz puncta in promoting amnioserosa AJ continuity is unclear. Their wide spacing suggests that any AJ scaffolding function would only affect a subset of cadherin-catenin complexes, although lower levels of more disperse Baz could provide this activity. In addition, mammalian Par-3 docks the exocyst complex (Ahmed and Macara, 2017) and regulates cortical actin networks (Chen and Macara, 2005), activities that could promote AJ continuity during growth and maintenance of large amnioserosal cell circumferences. In U-shaped-group mutants, amnioserosal fragmentation has been attributed to amnioserosal apoptosis (Frank and Rushlow, 1996), but in Baz RNAi embryos, amnioserosal cells with AJ fragmentation display no detectable abnormalities of their nuclei (Fig. S4), plasma membranes (Fig. S4) or MT networks (Fig. 7A). Overall, amnioserosal AJ continuity depends more on Baz than on α-catenin, and distinctive Baz puncta along amnioserosal AJs may be involved.

Amnioserosal cells gain unique cell shapes and cell-cell interactions. Amnioserosal cells are spindle-shaped when viewed from the embryo surface and also from the side (Fig. 4A). Long-range curvilinearity of AJ-mediated cell-cell contacts is supported by MT bundles along their lengths (Fig. 3A-C). Viewed from the embryo surface, amnioserosal cell-cell contacts often bulge at the middle of the cell because of the nucleus. However, many amnioserosal nuclei are positioned below the tapered ends of neighbouring cells (Fig. 4B,C). This partial overlap allows packing of amnioserosal cells into a smaller space than could be achieved by a non-overlapping, tiled arrangement. Confinement of the amnioserosa is also evident from its outward bulging in our live imaging (Fig. 5D), and in samples observed by scanning electron microscopy (Turner and Mahowald, 1977). Moreover, laser cutting experiments indicate that its junctions experience compressive stress (Fig. 5A-C) and implicate MT bundles in bearing the stress (Fig. S7). We propose that MT bundles form cortical cages around the circumferences of amnioserosal cells, and that their flexural rigidity bears compressive stress (Kikumoto et al., 2006). Pushing the sides of MT bundles against cell-cell contacts might generate curvilinear shapes in some cells and could drive nuclei inward in others. When excessively broad expanses of amnioserosa form in *fog* mutants, fewer nuclei are driven inward (Fig. S8B), suggesting that normal germband positioning is responsible for compressing the amnioserosa. In other epithelia, compression leads to extrusions or delaminations of cells (Eisenhoffer et al., 2012; Marinari et al., 2012). In the fully elongated amnioserosa, confinement by the neighbouring germband appears to generate an arrangement of spindle-shaped cells with partial overlap and alignment.

How do amnioserosal cells become aligned? Planar polarization of cell-cell contacts within a tissue is one way to align epithelial cells (Devenport, 2014). As amnioserosal cells begin to elongate, they do display planar polarized actomyosin networks along extending cell-cell contacts, but their narrower tips lack coordinated movement as the initially columnar amnioserosal cells rotate into the plane of the tissue in one direction or the other (Pope and Harris, 2008). It also appears that planar polarity of cell-cell contacts is neither sufficient nor necessary for amnioserosal cell alignment. Planar polarity mechanisms are unable to align amnioserosal cells when the tissue elongates against an immobile germband, as occurs when the germband dissociates in embryos depleted of α-catenin (Fig. 1B,C) or of Armadillo/β-catenin (Pope and Harris, 2008), and when the germband lacks positional information in *bcd nos tsl* triple mutants (Pope and Harris, 2008). Planar polarity mechanisms are also unable to align amnioserosal cells across abnormally broad areas of amnioserosa tissue, as occurs when the germband extends off-course in *fog* mutants (Fig. 6B; Fig. S8B), and when amnioserosal tissue is induced around the entire embryo in *dorsal* mutants (Pope and Harris, 2008). Moreover, planar polarity mechanisms are linked to cell-cell contacts (Devenport, 2014), but AJ continuity is not needed for amnioserosal cells to retain global alignment of their microtubule networks in Baz RNAi embryos with amnioserosal AJ fragmentation and an intact surrounding germband (Fig. 7A). Instead of junctional planar polarity within the amnioserosa, confinement by the surrounding germband appears to be key for the global alignment of spindle-shaped amnioserosal cells. Our results are consistent with effects of confinement on nematically-organized, spindle-shaped cells in culture (Duclos et al., 2018, 2017, 2014). In particular, the amnioserosa gains +1/2 and −1/2 topological defects when it develops with abnormally high or low confinement. These defects are characteristic of nematically-organized systems (Balasubramaniam et al., 2022; Saw et al., 2018). Moreover, live imaging of α-catenin RNAi embryos shows amnioserosal cells locally twisting around each other as they attempt to elongate against an immobilized germband (Fig. S8A), a behaviour resembling that of MT bundles extending under confinement *in vitro* (Opathalage et al., 2019). Although contributions of amnioserosal planar polarity cannot be fully excluded, it appears that spindle-shaped amnioserosal cells gain nematic alignment due to their confinement within a thin strip of the embryo surface.

What is the relevance of amnioserosal cell alignment? It arises as part of a reciprocal relationship between two tissues with distinct material properties. Neighbour exchanges are integral to germband extension (Bertet et al., 2004; Blankenship et al., 2006; Irvine and Wieschaus, 1994; Sun et al., 2017). Neighbour losses and gains, together with cell shape changes and low cell alignment, indicate that the germband converges, extends and rounds the posterior pole with liquid-like tissue behaviour (Wang et al., 2020). In contrast, the amnioserosa elongates with a maintenance of neighbour relationships, a high level of cell alignment, and extreme cell shape change (Pope and Harris, 2008). Thus, the amnioserosa appears to elongate as a deformable solid. Amnioserosa elongation is partly explained by extensile MT networks driving tissue-autonomous cell shape changes (Pope and Harris, 2008), but without germband extension, elongating amnioserosal cells twist around each other in a dorsal patch (Fig. 1B,C; Fig. S8A) (Pope and Harris, 2008). To gain its natural horseshoe-like shape, the amnioserosa requires the germband to flow around the posterior pole and over the dorsal surface, and resulting confinement appears to generate nematic alignment of amnioserosal cells. This reorganization of the amnioserosa also appears to affect the germband. Specifically, germband extension is abnormal in the absence of the reorganization, as evident in *zen* mutants lacking amnioserosa specification (Pope and Harris, 2008; Rushlow and Levine, 1990). Moreover, cell alignment of the fully elongated amnioserosa contributes mechanically to subsequent germband retraction (Lynch et al., 2013; McCleery et al., 2019). We propose a stepwise model of germband-amnioserosa reciprocity: (1) the solid-like amnioserosa undergoes autonomous cell shape changes which allow full extension of the fluid-like germband, (2) confinement of elongating amnioserosal cells by the extending germband promotes their alignment, and (3) the alignment of spindle-shaped amnioserosal cells facilitates retraction of the germband. Central to this model of reciprocity is nematic ordering of amnioserosal cells based on both tissue-autonomous and non-autonomous effects.

## MATERIALS AND METHODS

### Embryo staging and imaging

To analyze stages 10-11, embryos were collected from 5 h 20 m to 7 h 20 m after egg laying at 25°C. To analyze stages 6-8, embryos were collected from 3 h 00 m to 3 h 50 m after egg laying at 25°C. Fly stocks listed in Table S1. Embryos were collected from apple juice agar plates, washed three times with 0.1% Triton X-100, dechorionated with 50% household bleach for 4 min on a nutator, and then washed three times with 0.1% Triton X-100.

For fixed imaging, embryos were fixed in a 1:1 solution of 3.7% formaldehyde/heptane (methanol-popped embryos) or 10% formaldehyde/heptane for 25 min (hand-peeled embryos). Methanol-popped embryos were devitellinized by shaking for 30 s. Hand-peeled embryos were devitellinized manually with insect pins. Embryos were blocked for 1.5 h with 1% normal goat serum/0.1% Triton X-100/PBS, and then nutated overnight at 4°C with primary antibodies diluted in 1% normal goat serum/0.1% Triton X-100/PBS. Embryos were then washed three times with 0.1% Triton X-100/PBS, blocked for 30 min with 1% normal goat serum/0.1% Triton X-100/PBS and nutated at room temperature for 2 h with secondary antibodies diluted in 1% normal goat serum/0.1% Triton X-100/PBS. Embryos were then washed three times with 0.1% Triton X-100/PBS before mounting in Aqua Polymount (Polysciences). DAPI and Phalloidin were added during secondary antibody incubations. Antibodies, fluorescent markers and dilutions are listed in Table S1. For live imaging, dechorionated embryos were dried, glued to a coverslip using embryo glue (tape dissolved in heptane) and covered in halocarbon 700 oil (Halocarbon products). This coverslip was mounted onto a glass-bottom culture dish with its original coverslip removed.

All imaging was carried out at room temperature. Most imaging was carried out on a Ti2 inverted microscope (Nikon) with a CSU-X1 spinning disk (Yokogawa), a Prime 95B sCMOS camera (Teledyne Photometrics), a 60× Plan Apochromat lambda NA 1.4 oil-immersion objective (Nikon) or a 100× Plan Apochromat VC NA 1.4 oil-immersion objective, and NIS-elements software (Nikon). Some imaging was conducted on a spinning disk confocal system from Quorum technologies with a 63× Plan Apochromat NA 1.4 objective (Carl Zeiss), an EM CCD camera (Hamamatsu Photonics) and Volocity 4 software (Quorum Technologies). Laser ablations were carried out using an Optimicroscan UV galvo scanner (Nikon) using a 355 nm, 1000 Hz UV laser at three stimulation points concentrated on the same junction. Data were collected in *z*-stacks with 0.3 µm step sizes.

### Post-acquisition analyses

Analyses were performed using Fiji (https://fiji.sc/) on datasets with at least two biological replicates. All graphs were plotted in Excel (Microsoft). Quantifications focused on amnioserosal cells only in contact with other amnioserosal cells and excluded amnioserosal cells with cell-cell junctions with germband cells.

Fluorescence intensities along junctions were assessed with line scans. We analyzed 1.5 µm projections using a 0.54 µm wide freehand line. A line of 15 µm length was drawn and the line intensity profile was plotted. Line intensities were not background subtracted nor normalized before plotting.

Fluorescence intensities at junctions were assessed with a 1.28 µm diameter circle. Measurements were background corrected by subtracting nearby cytoplasmic signal. In each embryo, five measurements per tissue were averaged. For myosin quantifications, measurements were normalized to the average germband signal per embryo. For assessments of degree of RNAi knockdown, measurements were normalized to the average germband intensity of co-stained Histone-RFP control embryos for each slide assessed.

Tortuosity of a junction was measured in 1.5 µm projections as the ratio of the actual length of a junction, including local undulations, divided by the long-range, smooth curvilinear distance between the same start and end points. The actual lengths of junctions were traced with the freehand line tool. The long-range, smooth curvilinear distance was determined with multiple, straight 5 µm lines drawn sequentially along the overall junction using the segmented line tool. Five junctional measurements were taken for each embryo and averaged to produce a single value per embryo for graphical and statistical comparisons.

The percentage of a projected nucleus area within the bounds of AJs of its cell was calculated after determining the total area of the projected nucleus and the area of the projected nucleus within surrounding AJs. When a nucleus was below multiple cell-cell junctions, the AJs of its cell were assumed to be those surrounding the greatest area of the nucleus at its apical-most confocal section.

Particle image velocimetry analyses were carried out using the IterativePIV plugin in Fiji. We compared 2.7 µm projections at time points 0.8 s before ablation and 4.8 s after ablation. Regions of interest (ROIs) of 23.8 µm by 23.8 µm (130 pixels by 130 pixels) or of 73.3 µm by 27.5 µm (400 pixels by 150 pixels) were selected for germband and amnioserosal images, respectively. Each ROI was processed with Gaussian blurring (sigma setting 1.5) and binarized through Auto Local Thresholding (Otsu algorithm and radius of 60 pixels). Vector fields were generated with the IterativePIV plugin using a correlation threshold of 0.95 and a search window of 32 pixels (5.9 µm) and an interrogation window of 16 pixels (2.9 µm). The resulting vector fields were imported into Excel. The *xy* coordinates of the start and end of each vector were compared with *xy* coordinates of the ablation site of each image to calculate the angle between the vector and the ablation site using the law of cosines. For non-ablated control embryos, the centre of the ROI was used as a reference point (see Fig. S5A,B). Vectors in the lowest 10% of magnitudes were removed from each analysis to avoid noise. Angles and magnitudes of remaining vectors were compared.

Nematic order quantifications were performed on 1.5 µm projections of 29.3 µm by 29.3 µm (160 pixel by 160 pixel) ROIs. For greater sampling, three distinct ROIs were quantified in each embryo (and sometimes had partial overlap): an ROI at the centre of the amnioserosa in the field of view, an ROI with the least cell alignment detected by eye and an ROI with the most cell alignment detected by eye. ROIs were binarized using Auto Local Thresholding, the NIBlack algorithm, and radii of 60 and 15 pixels for DE-cad and tubulin images, respectively. Non-junctional signals were manually deleted from DE-cad images. Vector fields were obtained with the OrientationJ plugin (local window of 4 pixels for DE-cad, local window of 6 pixels for MTs, and grid size of 10 vectors; see Fig. S5C). Vector field outputs were exported to Excel (Microsoft) and nematic order (Q) was calculated in Duclos et al. (2014) (equation in Fig. S5D). Other quantifications were carried out through simple manual counts or measurements.

### Statistical analyses

Due to non-normal data distributions, statistical comparisons were carried out using Mann–Whitney *U*-tests (www.socscistatistics.com/tests/mannwhitney/). *N* values refer to embryo numbers unless otherwise specified.

### Figure preparation

Graphs were prepared in Excel (Microsoft). Images were prepared using Fiji and Photoshop (Adobe). Figures were assembled in Photoshop (Adobe).

## Supplementary Material



10.1242/develop.202577_sup1 Supplementary information
