# Supplementary material for: Confinement promotes nematic alignment of spindle-shaped cells during Drosophila embryogenesis
Source: Development. 2024 Jun 28;151(13):dev202577. doi: 10.1242/dev.202577 (PMC11234378; doi:10.1242/dev.202577)
Supplement: Supplementary information [file develop-151-202577-s1.pdf]

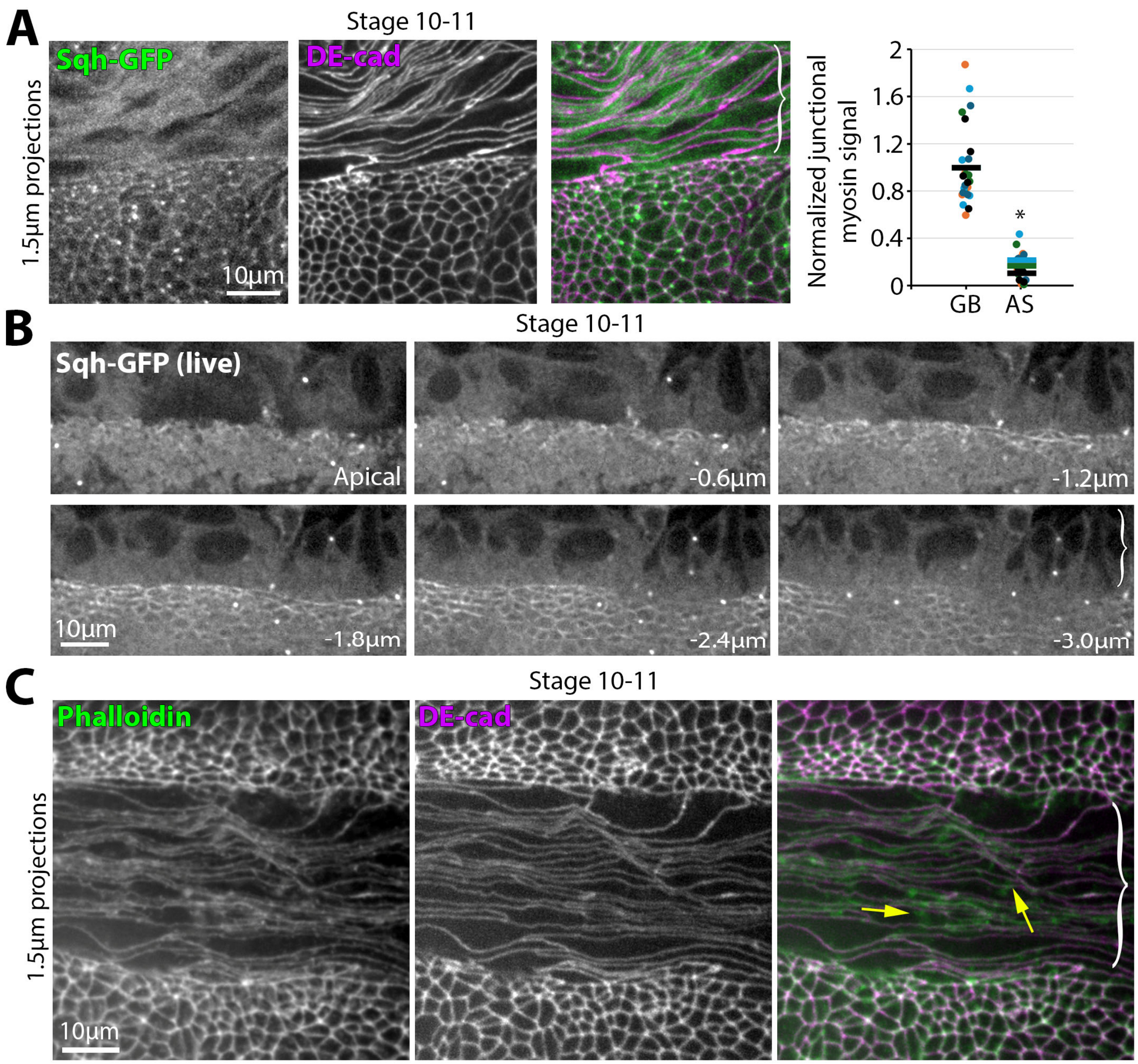

Fig. S1. Myosin and F-actin distributions of the amnioserosa are distinct from those of the germband. Related to Figure 1. (**A**) Stage 10-11 Sqh-GFP embryo stained for DE-cad. Bracket indicates amnioserosa. Junctional myosin signals quantified to right (amnioserosa, AS; germband, GB; asterisk, $p<0.05$ [comparing 5 embryos each]). Each dot represents one background-corrected fluorescence intensity measurement, with 5 measurements normalized to the germband average signal per embryo (each embryo is one colour). Bars represent averages of 5 measurements per embryo (germband averages overlap due to normalization). (**B**) Live stage 10-11 Sqh-GFP embryo showing myosin signals at multiple slices of a Z-stack. Bracket indicates amnioserosa. Difference in junctional myosin between the amnioserosa and germband seen in 16/16 embryos. (**C**) Stage 10-11 *yw* embryo stained with phalloidin and for DE-cad. White bracket indicates amnioserosa. Yellow arrows indicate distinct F-actin distribution in the amnioserosa. F-actin was less restricted to amnioserosal AJs compared to germband AJs in 14/14 embryos.

Development • Supplementary information

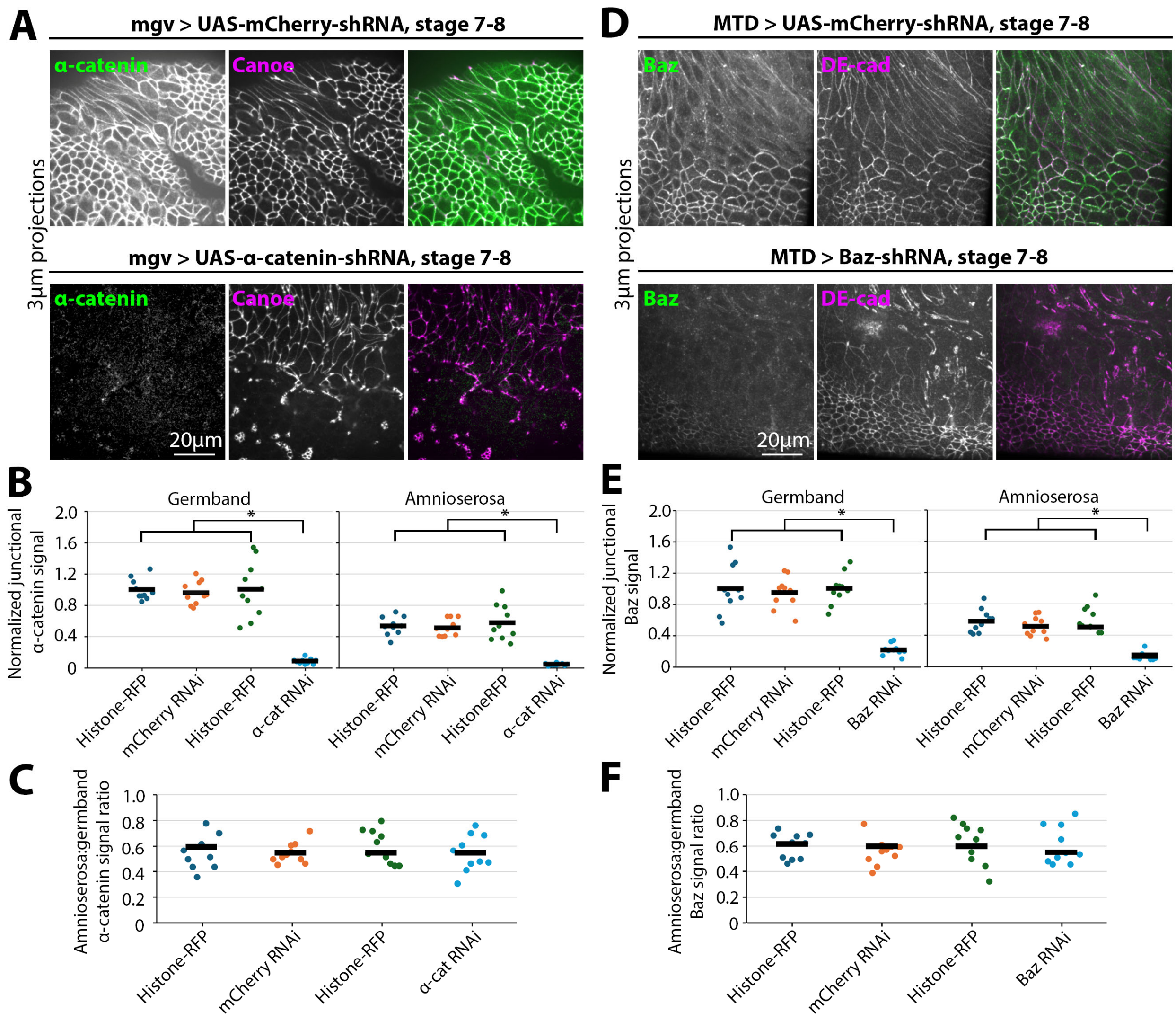

Fig. S2. Levels of α-catenin and Baz in the amnioserosa and germband following maternal shRNA expression. Related to Figures 1 and 2. (**A**) Projections of stage 7-8 mCherry RNAi and α-catenin RNAi embryos stained with α-catenin and with Canoe to mark AJs. shRNAs expressed maternally with maternal-α4-tubulin-GAL4::VP16 (mgv). Images collected and adjusted with the same settings. (**B**) Quantifications were normalized to the average values of co-stained Histone-RFP embryos which acted as internal references for each slide (mCherry RNAi and α-catenin RNAi). Each dot represents the mean of five measurements per embryo, and black bars represent the averages of all embryos per group. In the germband, cells with similar junctional circumferences were measured across genotypes to account for the tissue disruption with α-catenin RNAi. Asterisks indicate significant differences between the α-catenin RNAi embryos and all other genotypes ($p<0.001$, N=10 embryos per group). (**C**) Amnioserosa-to-germband ratios of junctional α-catenin signal were indistinguishable across the genotypes quantified in (B), indicating a similar degree of RNAi depletion of α-catenin in each tissue. Each dot is the ratio of one embryo. Black bars represent the averages of all embryos per group (N=10 embryos per group). (**D**) Projections of stage 7-8 mCherry RNAi and Baz RNAi embryos stained

with Baz and with DE-cad to mark AJs. shRNAs expressed maternally with Maternal Triple Driver (MTD). Images collected and adjusted with the same settings. (**E**) Quantifications were normalized to the average values of co-stained Histone-RFP embryos which acted as internal references for each slide (mCherry RNAi and Baz RNAi). Each dot represents the mean of five measurements per embryo, and black bars represent the averages of all embryos per group. Asterisks indicate significant differences between the Baz RNAi embryos and all other genotypes ($p<0.001$, N=10 embryos per group). (**F**) Amnioserosa-to-germband ratios of junctional Baz signal were indistinguishable across the genotypes quantified in (E), indicating a similar degree of RNAi depletion of Baz in each tissue. Each dot is the ratio of one embryo. Black bars represent the averages of all embryos per group (N=10 embryos per group).

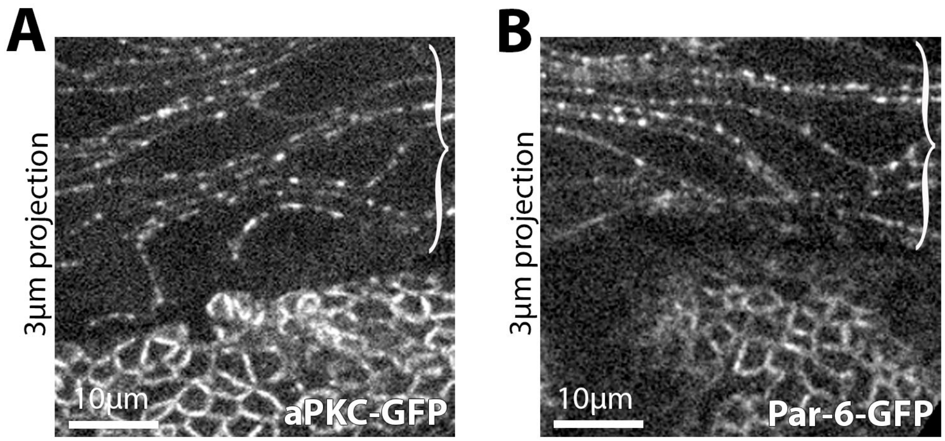

Fig. S3. Comparisons of aPKC and Par-6 distributions between the amnioserosa and germband. Related to Figure 2. (**A-B**) Live-imaged stage 10-11 embryos with distinctive aPKC-GFP (**A**) and Par6-GFP (**B**) puncta in the amnioserosa (brackets) compared to germband below. Seen in 8/8 aPKC-GFP embryos, and 9/9 Par6-GFP embryos.

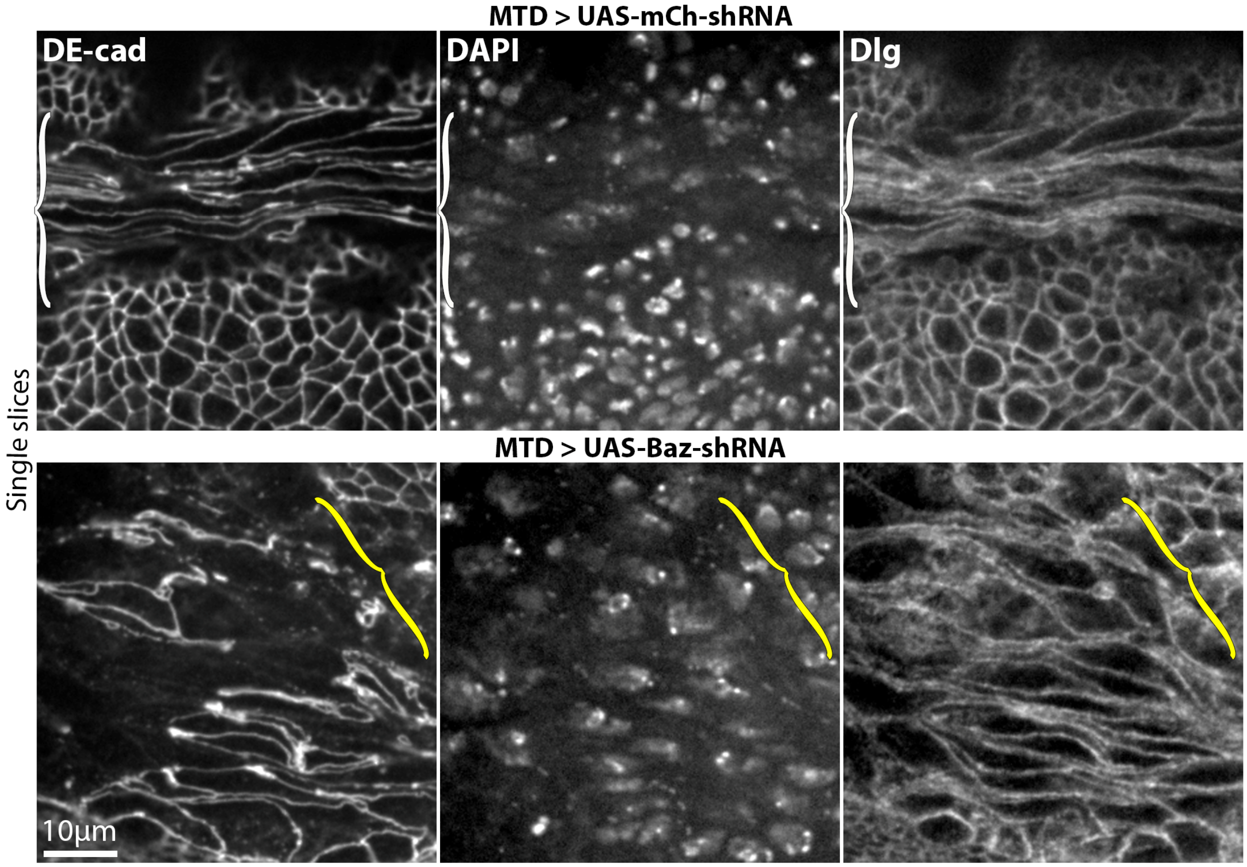

Fig. S4. Nuclei and basolateral membranes of amnioserosal cells seem unaffected in Baz RNAi embryos. Related to Figure 2. Stage 10-11 mCherry RNAi and Baz RNAi embryos stained with DAPI, Dlg, and DE-cad. Region of DE-cad loss in Baz RNAi amnioserosa (yellow brackets) displays indistinguishable DAPI and Dlg staining compared with region of continuous DE-cad junctional staining in mCherry RNAi amnioserosa (white brackets). Seen in 12/12 mCherry RNAi embryos and 11/11 Baz RNAi embryos.

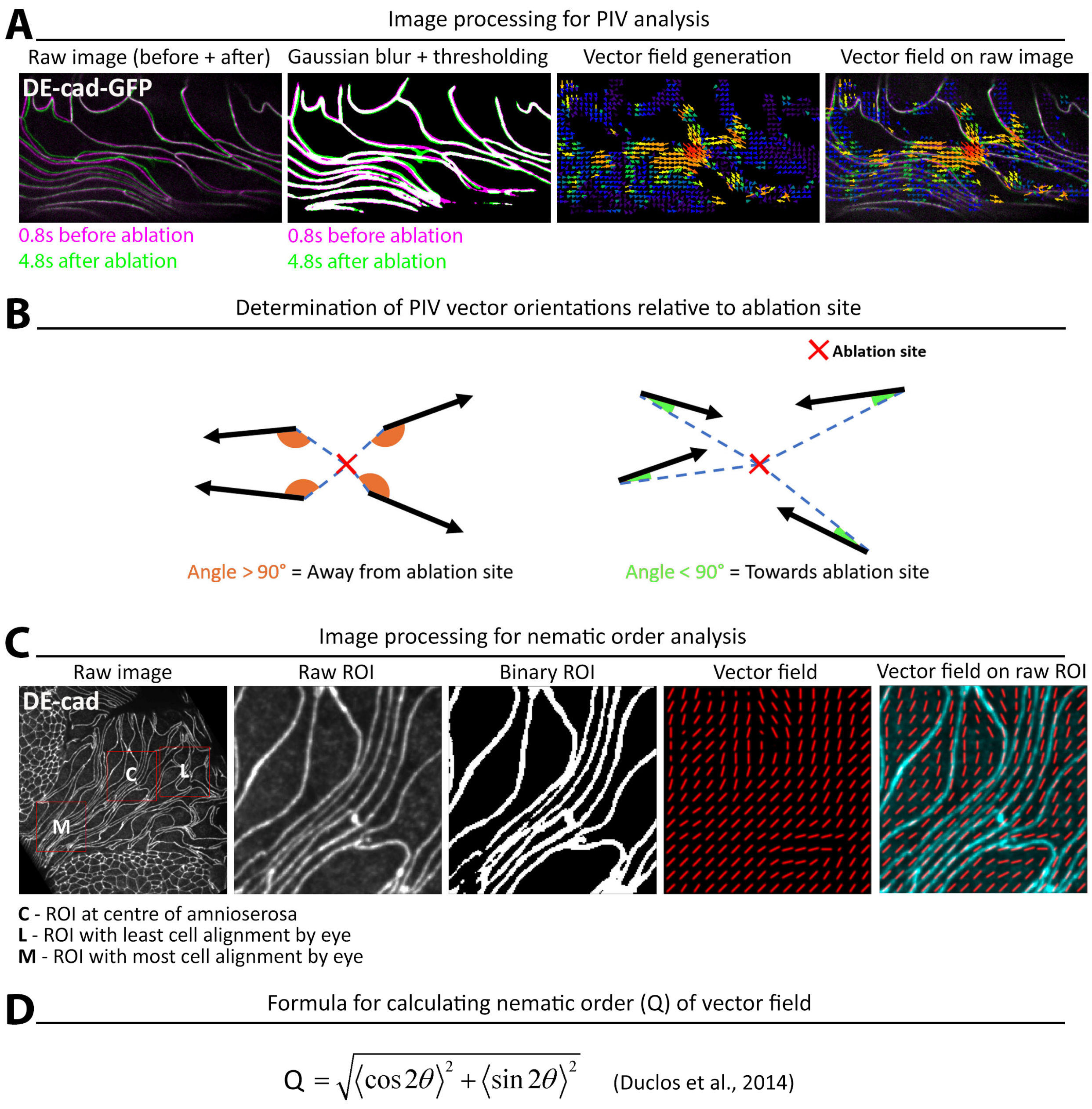

Fig. S5. Image processing workflow for PIV analyses and nematic order analyses. Related to Figures 5, 6 and 7. (**A**) A live DE-cad-GFP embryo with an amnioserosal ablation shown as an example of the imaging processing performed for PIV analyses. At left, the raw image showing the before (magenta) and after (green) time points used for the analysis. Each time point was then processed with a Gaussian blur and converted to binary from which a vector field was generated (see Methods). At left, an overlay of the vector field on the original image shows that the calculated vectors correspond with AJ movements upon ablation. (**B**) Schematics show how angles of the displacement vectors were determined in relation to the ablation site. Vectors moving away from the ablation site are compared are shown on the left, with those moving toward the ablation site are shown on the right. (**C**) A DE-cad-stained *fog* mutant embryo shown

as an example of the imaging processing performed to analyze nematic order. At left, the three distinct ROIs evaluated are indicated: the centre of the amnioserosa tissue imaged (C), the amnioserosal region with the least alignment detected by eye (L), and the amnioserosal region with the most alignment detected by eye (M). Each ROI was then cropped and converted to a binary image from which a vector field was generated (see Methods). At right, an overlay of the vector field on the original image shows that the calculated vectors correspond to local AJ orientations. (**D**) Equation used to calculate nematic order (Q) of the vector field.

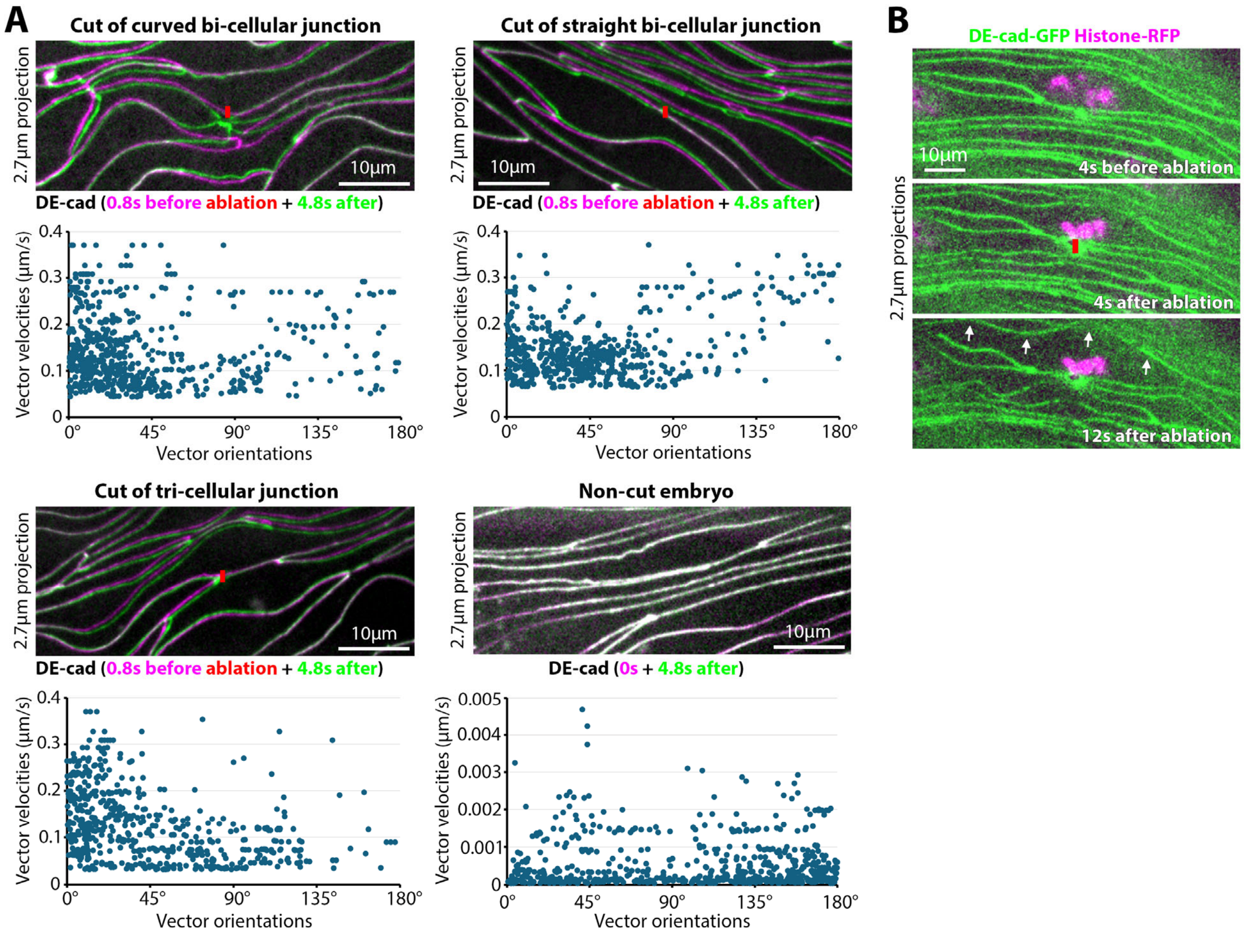

Fig. S6. Amnioserosal laser ablations result in consistent tissue-wide movements and occasional local effects. Related to Figure 5. (**A**) Amnioserosal ablations of curved bi-cellular junctions, straight bi-cellular junctions, or tri-cellular junctions all result in most surrounding junctions moving toward the cut site. Responses of individual embryos shown and quantified below. An example of a non-cut embryos with minimal junctional movement is also shown and quantified. (**B**) An embryo co-expressing DE-cad-GFP and Histone-RFP shows an outward expansion of cell-cell contacts (arrows) correlated with cutting and structural change to the nucleus of the cell.

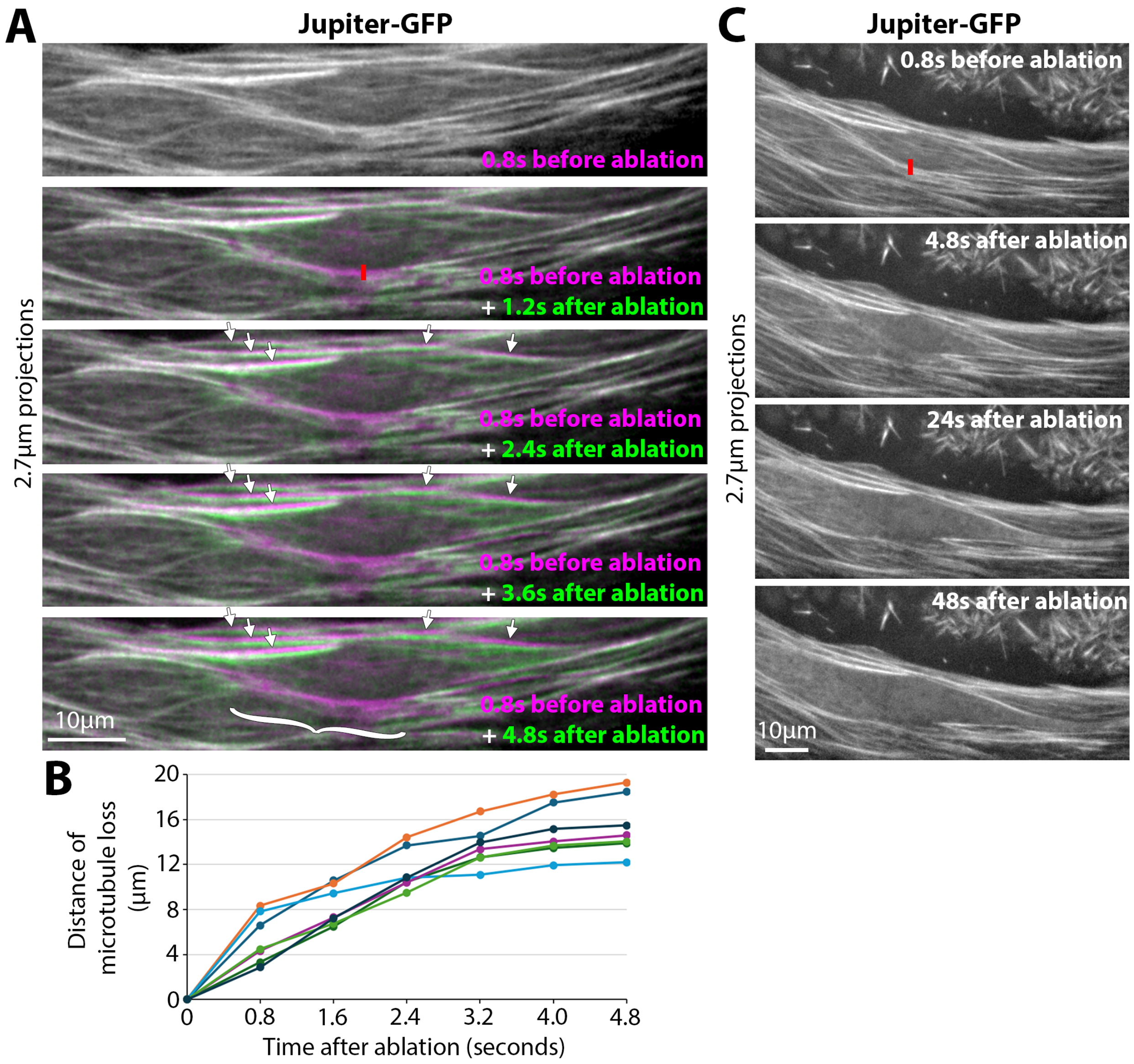

Fig. S7. Responses of amnioserosal MT bundles to local ablation. Related to Figure 5. (**A**) A stage 10-11 Jupiter-GFP embryo ablated at a MT bundle along an amnioserosal cell-cell contact. Red line indicates ablation site. Bracket indicates depolymerization of ablated MT bundles away from the ablation site. Arrows indicate shifts of surrounding MT bundles toward the ablation site (seen in 5/7 embryos). (**B**) Quantification of the distance of ablated MT bundle loss over time. Responses of 7 embryos plotted. (**C**) The ablation-induced MT disassembly expands through the whole cell over time. Red line indicates ablation site.

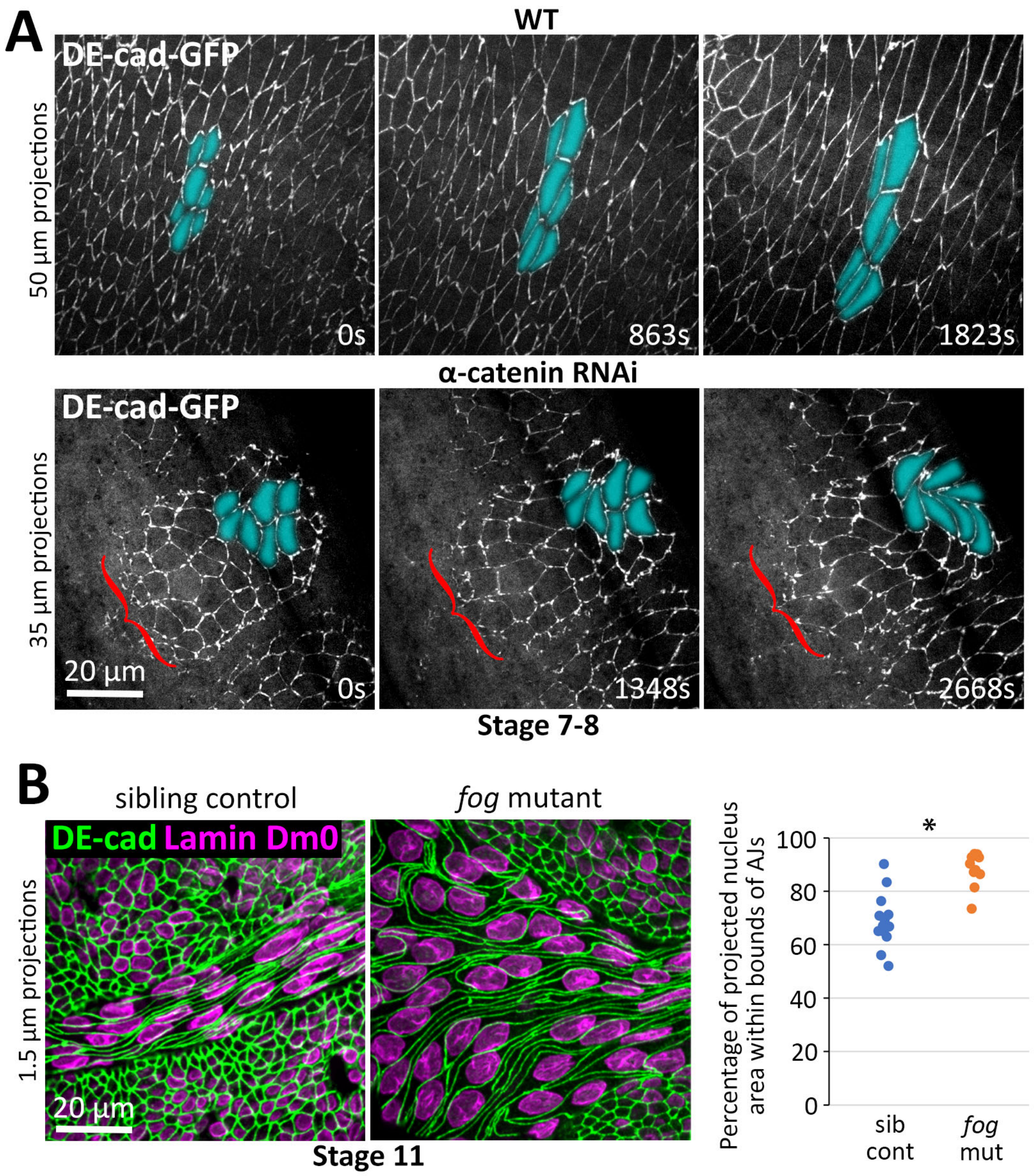

Fig. S8. Evidence of increased or decreased amnioserosal confinement with distinct disruptions of the surrounding germband. Related to Figures 6 and 7. (**A**) In stage 7-8 DE-cad-GFP controls, individual amnioserosal cells elongate in alignment (seven cells coloured in cyan) as the whole amnioserosal tissue elongates. In stage 7-8 α-catenin RNAi embryos expressing DE-cad-GFP, individual amnioserosal cells elongate but become misaligned (seven cells coloured in cyan) as elongation of the whole amnioserosal tissue seems blocked by the dissociated germband (red brackets indicate the boundary between the tissues). Imaging of early amnioserosa elongation showed cell alignment in 6/6 control embryos and mis-alignment in 5/5 α-catenin RNAi embryos. (**B**) Stage 10-11 *fog* mutants and sibling controls stained with Lamin Dm0 and DE-cad. In *fog* mutants, projected nuclei areas extend less beyond the bounds of overlying AJs. Graph shows corresponding quantification and significant difference ($p<0.05$; 13 sibling control embryos; 10 *fog* mutant embryos).

Development • Supplementary information

Table S1. Drosophila stocks and molecular probes

Reagent	Source	Reference
1. Fly strains		
1.1 Mutant alleles		
fog^{S4}	Bloomington Drosophila Stock Center (BDSC) #2100	(Wieschaus et al., 1984)
yellow white (*yw*) (general control)	Mark Peifer UNC Chapel Hill, USA	None
1.2 Proteins expressed by native promoters		
aPKC-GFP	Francois Schweisguth Pasteur Institute, France	(Besson et al., 2015)
Baz-GFP	FlyTrap Project	(Kelso et al., 2004)
DE-Cad-GFP	Yang Hong University of Pittsburgh, USA	(Huang et al., 2009)
DE-Cad-RFP	Yang Hong University of Pittsburgh, USA	(Huang et al., 2009)
Histone-GFP	BDSC #24163	(Crest et al., 2007)
Histone-RFP	BDSC #23650	(Schuh et al., 2007)
Jupiter-GFP	BDSC#6836	(Morin et al., 2001)
Par6-GFP	Juergen Knoblich IMBA, Austria	(Wirtz-Peitz et al., 2008)
Spider-GFP	BDSC #59025	(Morin et al., 2001)
Sqh-GFP	Roger Karess Institut Jacques Monod, France	(Royou et al., 2002)
1.3 Proteins expressed by non-native promoters		
PH-mCherry	Yohanns Bellaiche Curie Institute, France	(Herszterg et al., 2013)
Tubulin-GFP	Allan Spradling Carnegie Institution of Washington, USA	(Grieder et al., 2000)
UAS-Baz-GFP	Our lab	(McKinley et al., 2012)
UAS-BazΔOD-GFP	Our lab	(McKinley et al., 2012)
UAS-Spastin	Nina Sherwood Duke University, USA	(Sherwood et al., 2004)
1.4 UAS shRNA lines		
UAS-Baz-shRNA (Baz 39072)	BDSC #39072	(Perkins et al., 2015)
UAS-Baz-shRNA (Baz 9)	Our lab	(Jiang et al., 2015)
UAS-mCh-shRNA	BDSC #35785	(Perkins et al., 2015)
UAS-α-catenin-shRNA (33430)	BDSC #33430	(Perkins et al., 2015)
UAS-α-catenin-shRNA (38987)	BDSC #38987	(Perkins et al., 2015)
1.5 GAL4 lines		
Daughterless-GAL4 (daGAL4)	Elisabeth Knust	(Wodarz et al., 1995)

	Max Planck Institute of Molecular Cell Biology and Genetics, Germany	
Maternal-α4-tubulin-GAL4::VP16 (mgv)	Mark Peifer<br>UNC Chapel Hill, USA	None
Maternal Triple Driver (MTD)	BDSC #31777	None
2. Antibodies/Fluorescent markers		
DAPI<br>1:1000 dilution	Sigma-Aldrich	Cat#D9542
Goat anti-mouse Alexa Fluor 568<br>1:500 dilution	Invitrogen	Cat#A11004
Goat anti-rat Alexa Fluor 647<br>1:500 dilution	Invitrogen	Cat#A48265
Mouse anti-Discs large (4F3)<br>1:100 dilution	Developmental Studies Hybridoma Bank (DSHB)	(Parnas et al., 2001)
Mouse anti-Lamin Dm0 (ADL67.10)<br>1:100 dilution	DSHB	(Riemer et al., 1995)
Mouse anti-Tubulin (E7)<br>1:100 dilution	DSHB	(Chu and Klymkowsky, 1989)
Phalloidin (Alexa Fluor 568)<br>1:200 dilution	Invitrogen	Cat#A-12380
Rabbit anti-Baz<br>1:3500 dilution	Our lab	(McGill et al., 2009)
Rabbit anti-Cno<br>1:500 dilution	Mark Peifer<br>UNC Chapel Hill, USA	(Sawyer et al., 2011)
Rat anti-α-catenin (DCAT1)<br>1:100 dilution	DSHB	(Oda et al., 1993)
Rat anti-DE-cad (DCAD2)<br>1:100 dilution	DSHB	(Oda et al., 1994)

Development • Supplementary information

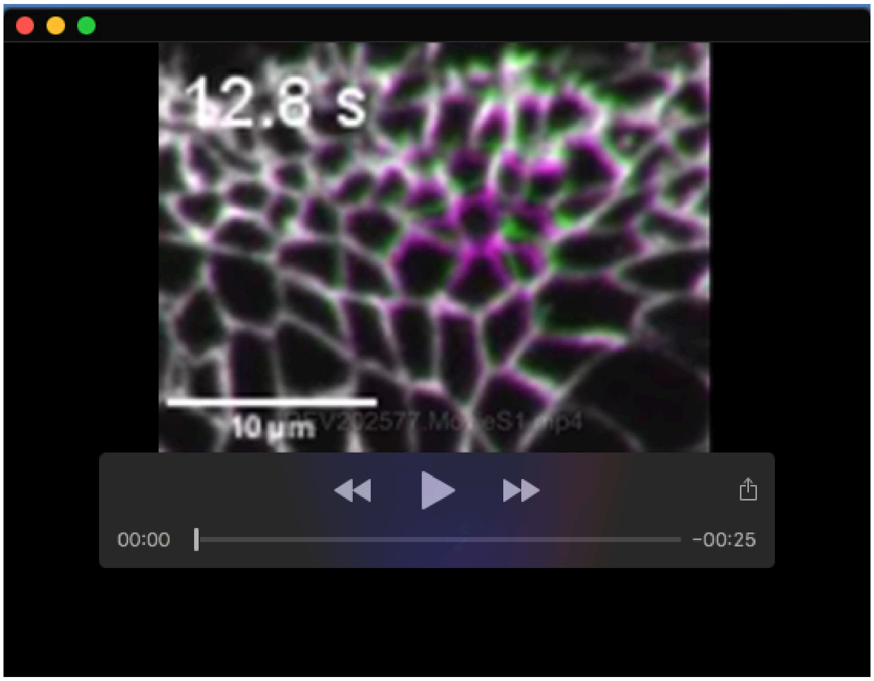

Movie 1. Live stage 10-11 DE-cad-GFP embryo before (purple) and after (green) laser ablation of a germband cell-cell junction. Tissue movement occurs away from the ablation site (seen in 7 embryos). Individual time points shown in Figure 5A.

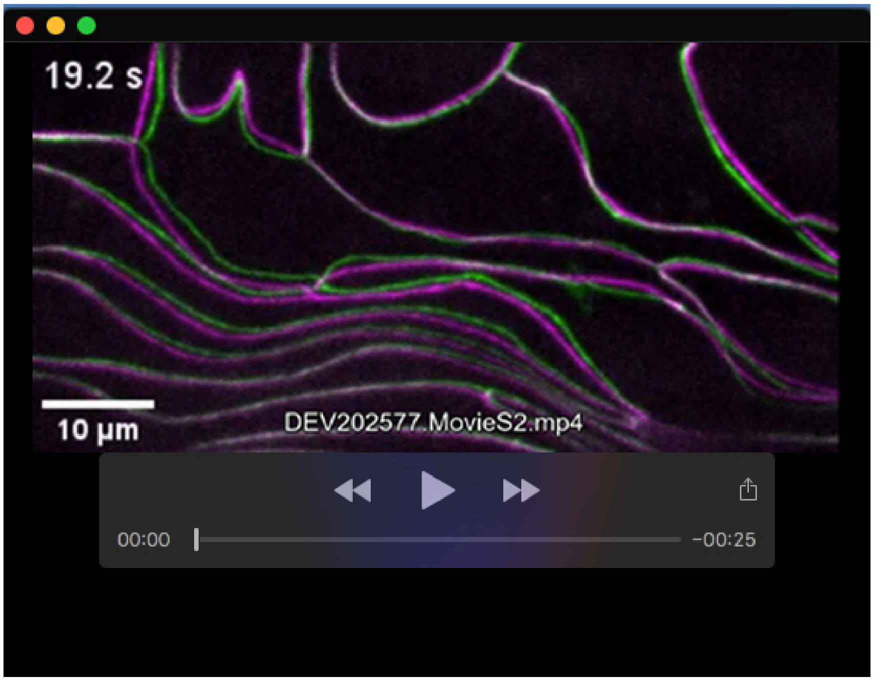

Movie 2. Live stage 10-11 DE-cad-GFP embryo before (purple) and after (green) laser ablation of an amnioserosal bi-cellular junction. Tissue movement occurs toward the ablation site. An example of a strong response shown (seen in bi-cellular and tri-cellular cuts of 17 embryos to various degrees). Individual time points shown in Figure 5A.

Development • Supplementary information

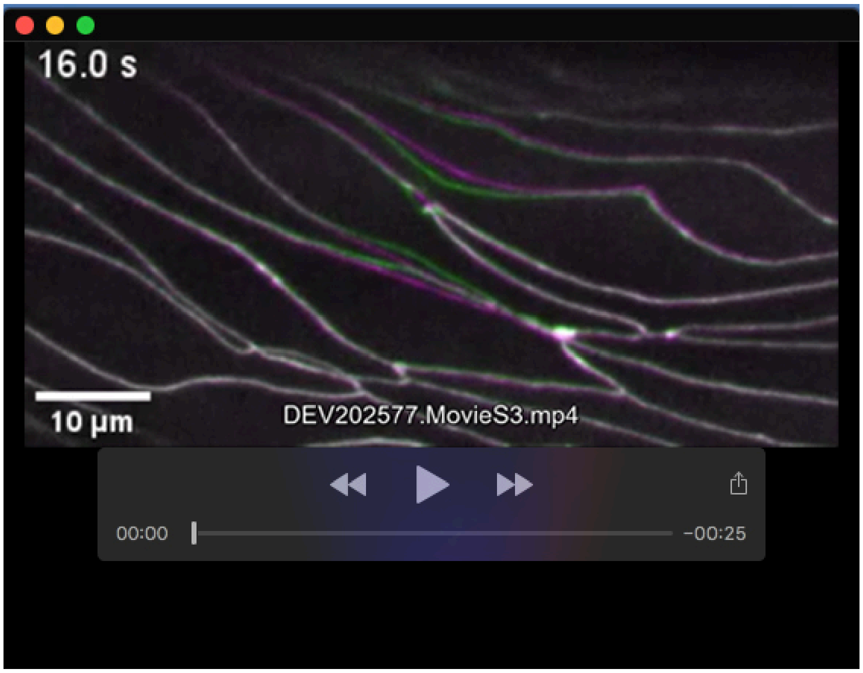

Movie 3. Live stage 10-11 DE-cad-GFP embryo before (purple) and after (green) laser ablations of an amnioserosal tri-cellular junction. Tissue movement occurs toward the ablation site. An example of a weak response shown (seen in bi-cellular and tri-cellular cuts of 17 embryos to various degrees).

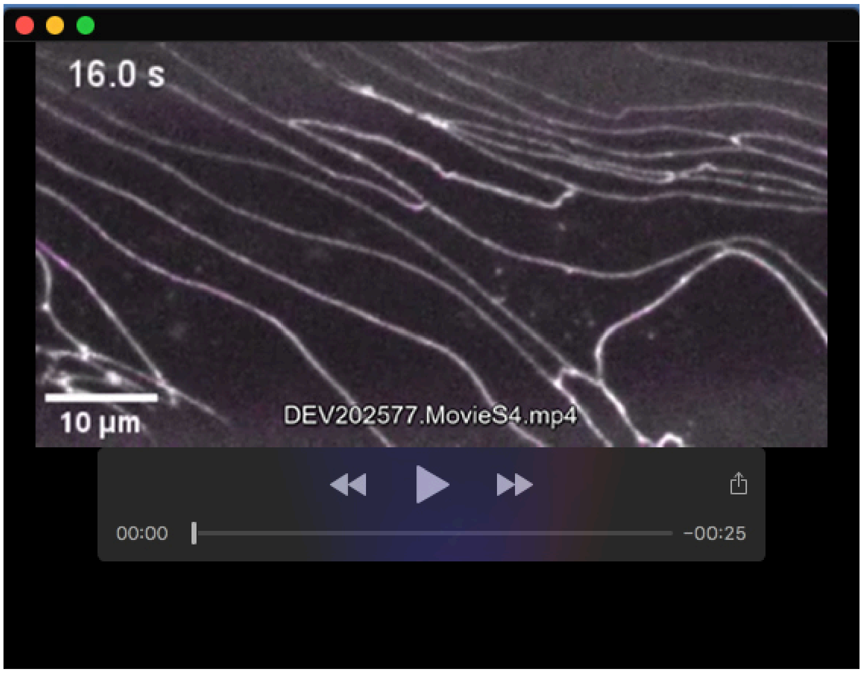

Movie 4. Live stage 10-11 DE-cad-GFP embryo imaged without ablation. Live imaging shows lack of movement in the amnioserosa without laser ablations (seen in 5 embryos). The signal at the start of imaging (purple) is overlaid with the signals of the following time points (green). The original purple signal becomes dominant over time as the sample bleaches.

Development • Supplementary information
