# Supplementary material for: Confinement promotes nematic alignment of spindle-shaped cells during Drosophila embryogenesis
Source: Development. 2024 Jun 28;151(13):dev202577. doi: 10.1242/dev.202577 (PMC11234378; doi:10.1242/dev.202577)
Supplement: Supplementary file 1 [file develop-151-202577_review_history.pdf]

Development

Confinement promotes nematic alignment of spindle-shaped cells during *Drosophila* embryogenesis

Tirthankar Ray, Damo Shi and Tony J.C. Harris
DOI: 10.1242/dev.202577

Editor: Thomas Lecuit

Review timeline
Original submission: 30 November 2023
Editorial decision: 14 February 2024
First revision received: 22 April 2024
Editorial decision: 28 May 2024
Second revision received: 4 June 2024
Accepted: 5 June 2024

Original submission

First decision letter

MS ID#: DEVELOP/2023/202577

MS TITLE: Confinement promotes nematic alignment of spindle-shaped cells during Drosophila embryogenesis

AUTHORS: Tirthankar Ray, Damo Shi, and Tony Harris

I sincerely apologise for the delay in getting back to you due to one of the reviewers not returning their review. I have now received all the referees' reports on the above manuscript, and have reached a decision. The referees' comments are appended below, or you can access them online: please go to BenchPress and click on the 'Manuscripts with Decisions' queue in the Author Area.

As you will see, the referees express interest in your work, as do I, but have some criticisms and recommend a revision of your manuscript before we can consider publication. The two main issues are 1) the characterization of cell compression on AS cells and 2) the better documentation of knock down eg. using antibody staining. If you are able to revise the manuscript along the lines suggested, which will involve further experiments, I will be happy receive a revised version of the manuscript. Your revised paper will be re-reviewed by one or more of the original referees, and acceptance of your manuscript will depend on your addressing satisfactorily the reviewers' major concerns. Please also note that Development will normally permit only one round of major revision. If it would be helpful, you are welcome to contact us to discuss your revision in greater detail. Please send us a point-by-point response indicating your plans for addressing the referees' comments, and we will look over this and provide further guidance.

Please attend to all of the reviewers' comments and ensure that you clearly highlight all changes made in the revised manuscript. Please avoid using 'Tracked changes' in Word files as these are lost in PDF conversion. I should be grateful if you would also provide a point-by-point response detailing how you have dealt with the points raised by the reviewers in the 'Response to Reviewers' box. If you do not agree with any of their criticisms or suggestions please explain clearly why this is so.

Reviewer 1

© 2024. Published by The Company of Biologists under the terms of the Creative Commons Attribution License (https://creativecommons.org/licenses/by/4.0/).

Advance summary and potential significance to field

The manuscript by Ray et al. presents a set of experiments concerning an early phase of amnioserosa morphogenesis at the end of germband extension, when the cells are extremely elongated to spindle-shaped cells. Firstly, the authors present data about the distribution of F-actin and Baz along adherens junctions and their dependance on a-catenin. The authors claim that adherens junctions of the amnioserosa are less dependent on a-catenin but more sensitive to Baz depletion than in the epidermis based on a differential sensitivity to maternal depletion of a-catenin and Baz, respectively. Secondly, the authors describe microtubules oriented along the long axis of the extended amnioserosa cells and test the MT function by expression of the severing enzyme spastin. Cells remained extended but display less straight junctions. Thirdly, the authors present data supporting a pseudo-stratified structure of the AS at that stage. In the last three figures, the authors argue and present corresponding data that the amnioserosa would be under compression by the neighboring epidermis and that an ordered alignment would be an non-autonomous effect due to the epidermis. Finally, they present data showing that the structural dependencies concerning the adherens junctions described in the first two/three figures have no consequence for the alignment of the extend cells.

Comments for the author

The manuscript appears to me as a collection of interesting data with the amnioserosa as a common denominator. I do not recognize a clear line of argumentation, which would lead to a central conclusion. In addition, some of the claims or conclusions are not well supported by the presented data (differential RNAi phenotypes can be based on differential stabilities, expression or protein turnover). The quantification of the alignment of extended cells by an order parameter is nice, but the assay is not really used to come to a novel insight. I do not support publication of the manuscript in its current version.

• The introduction is rather long and elaborated. It partially appears more of a review of previous work rather than a succinct presentation of the necessary background leading to a defined question.

• Myosin: Already in the first sentence of the abstract myosin is mentioned. This is followed by statements that the AS cells lack junctional myosin. This goes on with a full paragraph about actomyosin at junctions in the introduction. Similarly the first sentence in the results section cites previous reports about low junctional myosin in the amnioserosa, which is used as a starting hypothesis that adherens junctions in the amnioserosa are different. After all this, I expected to see some myosin stainings or live imaging reproducing at least the previous data by state-of-the-art imaging including a quantification. I am also confused by the varying descriptions of “lack”, “minimal”and “low”. The conclusive statement of the first paragraph (l133) even includes myosin “.. display minimal myosin association…” I did not find any data about myosin in the manuscript.

• Differential RNAi phenotypes between AS and epidermis. The authors employ maternal RNAi for downregulation of a-catenin and baz. The authors assume a similar depletion of the target genes in the two distinct cell types/tissues, amnioserosa and epidermis. The authors do not present any data supporting the validity of their assumption such as a staining for a-catenin or baz as a control, at least. The different cell types may have differential turnover of the proteins, may have differential zygotic expression or even differential stability of the siRNAs. In my view, a conclusion about different structure and stability of adherens junctions is not supported by the data presented.

• AS as a pseudostratified tissue. In my view the presented data are a good starting point towards the conclusion but remain indirect and circumstantial. What is lacking is a direct demonstration of a multilayered structure by axial images reconstructed from an image stack or images from a cross section perpendicular to the embryonic axis, for example.

• Compression in the AS: These experiments appear anecdotal as they lack quantification. I can follow the description of the data by the authors, but I am puzzled by the wide-ranging effect in the amnioserosa over more than 50 µm, whereas the recoil effect is restricted to immediate

© 2024. Published by The Company of Biologists under the terms of the Creative Commons Attribution License (https://creativecommons.org/licenses/by/4.0/).

neighbors with less than 10 µm range in the epidermis. I find it difficult to interpret the data towards compression, if the read-out is done a minute after application of the laser flash. The mechanics (including compression) will be immediately visible at least within seconds. Later effects may include a biological response that effectively behaves like compression.

Reviewer 2

Advance summary and potential significance to field

Ray et al. examine the mechanisms that underlie the common orientation of amnioserosal (AS) cells in the Drosophila embryo. They hypothesize that compressive forces from the surrounding germband tissue, a low adhesion regime in the AS cells, and microtubule reinforcement at the boundaries of AS cell help this tissue align cells through a nematic process along the AP axis. The work is interesting and mostly well-done. A few major and minor comments are below:

Comments for the author

1) The laser cuts that are performed to suggest AS compression need much better documentation. This is a major point of the study, but is currently shown by just one example of a cut in a single panel. This needs to be shown in a much better way – before and after images, cuts in multiple places along cells (the one AS image looks like the cut was done in an inflection point in cell curvature), etc. Quantitation should be performed.

2) The Introduction is nicely written, with one exception. The authors say, “The most common topological defects associated with nematically ordered systems are comet-like +1/2 defects and threefold symmetric -1/2 defects”. There is zero definition of what this means – much greater effort needs to be done to explain this and the later quantitation. The one schematic is not particularly helpful.

3) It is assumed that aPKC and Par6 are in the same place as Bazooka because they are punctate. This should be shown – reagents exist to do these colocalization experiments.

4) Line 240 “data not shown”. Believe this is against Development’s data policies.

5) The authors should work on their figure layouts and do statistical testing in each circumstance where differences are reported (Fig. 2D, for example). The statistical test used should be reported in each figure legend, and appropriate parametric or non-parametric tests (depending on data type/distributions) used.

Minor notes

a) Would pay good money to whoever rips out all the bracket keys in the keyboards in the lab (kidding but kind of not. So many, so overused, so distracting).

b) And so… Are white brackets missing in Fig. S2?

First revision

Author response to reviewers' comments

Responses to reviews

Reviewer 1 Advance Summary and Potential Significance to Field:
The manuscript by Ray et al. presents a set of experiments concerning an early phase of amnioserosa morphogenesis at the end of germband extension, when the cells are extremely elongated to spindle- shaped cells. Firstly, the authors present data about the distribution of F-

© 2024. Published by The Company of Biologists under the terms of the Creative Commons Attribution License (https://creativecommons.org/licenses/by/4.0/).

actin and Baz along adherens junctions and their dependance on a-catenin. The authors claim that adherens junctions of the amnioserosa are less dependent on a-catenin but more sensitive to Baz depletion than in the epidermis based on a differential sensitivity to maternal depletion of a-catenin and Baz, respectively. Secondly, the authors describe microtubules oriented along the long axis of the extended amnioserosa cells and test the MT function by expression of the severing enzyme spastin. Cells remained extended but display less straight junctions. Thirdly, the authors present data supporting a pseudo-stratified structure of the AS at that stage. In the last three figures, the authors argue and present corresponding data that the amnioserosa would be under compression by the neighboring epidermis and that an ordered alignment would be an non-autonomous effect due to the epidermis. Finally, they present data showing that the structural dependencies concerning the adherens junctions described in the first two/three figures have no consequence for the alignment of the extend cells.

We thank the reviewer for this summary and for their helpful comments below.

Reviewer 1 Comments for the Author:
The manuscript appears to me as a collection of interesting data with the amnioserosa as a common denominator. I do not recognize a clear line of argumentation, which would lead to a central conclusion. In addition, some of the claims or conclusions are not well supported by the presented data (differential RNAi phenotypes can be based on differential stabilities, expression or protein turnover). The quantification of the alignment of extended cells by an order parameter is nice, but the assay is not really used to come to a novel insight. I do not support publication of the manuscript in its current version.

To clarify our line of argumentation and our overall conclusion, we have revised the paper at key transitions throughout. For example, in the Abstract we clarified our research question (Line 26: “What are the bases of amnioserosal cell interactions and alignment?”) and our overall conclusion (Line 37: “Overall, the fully elongated amnioserosa seems to form through tissue-autonomous generation of spindle-shaped cells which nematically align in response to confinement by surrounding tissue”. Other studies have shown the functional relevance of this alignment to germband development. We end the Discussion with the following synthesis (lines 431-436): “We propose a stepwise model of germband- amnioserosa reciprocity: (1) the solid-like amnioserosa undergoes autonomous cell shape changes which allow full extension of the fluid-like germband, (2) confinement of elongating amnioserosal cells by the extending germband promotes their alignment, and (3) the alignment of spindle-shaped amnioserosal cells facilitates retraction of the germband. Central to this model of reciprocity is nematic ordering of amnioserosal cells based on both tissue-autonomous and non-autonomous effects.”

In response to concerns about the data, we have conducted additional analyses, as described below.

•*The introduction is rather long and elaborated. It partially appears more of a review of previous work rather than a succinct presentation of the necessary background leading to a defined question.*

Revisions have focused the Introduction.

•*Myosin: Already in the first sentence of the abstract myosin is mentioned. This is followed by statements that the AS cells lack junctional myosin. This goes on with a full paragraph about actomyosin at junctions in the introduction. Similarly the first sentence in the results section cites previous reports about low junctional myosin in the amnioserosa, which is used as a starting hypothesis that adherens junctions in the amnioserosa are different. After all this, I expected to see some myosin stainings or live imaging reproducing at least the previous data by state-of-the-art imaging including a quantification. I am also confused by the varying descriptions of “lack”, “minimal”and “low”. The conclusive statement of the first paragraph (l133) even includes myosin “.. display minimal myosin association…” I did not find any data about myosin in the manuscript.*

Data and quantifications have been added to further document junctional myosin localization in the fully elongated amnioserosa. This localization was previously shown in fixed samples without quantification in Pope and Harris (2008). We now show the localization in fixed samples with accompanying quantifications, and also show the localization in live samples. Please see the

© 2024. Published by The Company of Biologists under the terms of the Creative Commons Attribution License (https://creativecommons.org/licenses/by/4.0/).

following text and data:

Line 135: “During formation and at full elongation, the amnioserosa has relatively low junctional myosin compared to the germband (Bertet et al., 2004; Pope and Harris, 2008), a difference we confirmed and quantified in the fully elongated tissue at stage 10-11 (Figure S1A-B).”

Revisions were also made to unify our descriptions of junctional myosin in the fully-elongated amnioserosa. Please see lines 26, 89, 139, and 205.

•Differential RNAi phenotypes between AS and epidermis. The authors employ maternal RNAi for downregulation of a-catenin and baz. The authors assume a similar depletion of the target genes in the two distinct cell types/tissues, amnioserosa and epidermis. The authors do not present any data supporting the validity of their assumption such as a staining for a-catenin or baz as a control, at least. The different cell types may have differential turnover of the proteins, may have differential zygotic expression or even differential stability of the siRNAs. In my view, a conclusion about different structure and stability of adherens junctions is not supported by the data presented.

Thank you for raising this important consideration. We assessed RNAi target depletion in both tissues by comparing control RNAi and target RNAi embryos, as well as co-stained Histone-RFP embryos as common internal controls in both samples. Our quantifications of these data showed that junctional levels of α-catenin and Baz were substantially reduced following their respective RNAi depletions. More specifically, amnioserosa:germband signal ratios were indistinguishable with or without the RNAi depletion, indicating that the RNAi depletion occurred to a similar degree in both tissues. Please see the following additions of text and data:

Line 145: “Staining for α-catenin showed that its junctional levels were depleted by a similar degree in both the germband and the amnioserosa (Figure S2A-C).”

Line 189: “Staining for Baz showed that its junctional levels were depleted by a similar degree in both the germband and the amnioserosa (Figure S2D-F).”

There were two caveats to these analyses. First, α-catenin RNAi causes disruption to the germband and germband cells that retain junctional α-catenin have abnormally small apicolateral circumferences. This issue was addressed by quantifying germband cells of similar sizes in α-catenin RNAi embryos, control RNAi embryos, and Histone-RFP (internal control) embryos (Figure S2 legend: “In the germband, cells with similar junctional circumferences were measured across genotypes to account for the tissue disruption with α-catenin RNAi.”). Second, the distribution of Baz in the amnioserosa is altered by fixation (Line 174: “The Baz puncta were not maintained with embryo fixation (Figure S2D)”). This issue was assumed to affect all embryos similarly (Baz RNAi embryos, control RNAi embryos, and Histone-RFP embryos).

•AS as a pseudostratified tissue. In my view the presented data are a good starting point towards the conclusion but remain indirect and circumstantial. What is lacking is a direct demonstration of a multilayered structure by axial images reconstructed from an image stack or images from a cross section perpendicular to the embryonic axis, for example.

To help clarify the multilayered tissue organization, reconstructed Z-sections have been added to the two distinct analyses implicating pseudostratification. Please see Figures 4B and 4C.

•Compression in the AS: These experiments appear anecdotal as they lack quantification. I can follow the description of the data by the authors, but I am puzzled by the wide-ranging effect in the amnioserosa over more than 50 µm, whereas the recoil effect is restricted to immediate neighbors with less than 10 µm range in the epidermis. I find it difficult to interpret the data towards compression, if the read-out is done a minute after application of the laser flash. The mechanics (including compression) will be immediately visible at least within seconds. Later effects may include a biological response that effectively behaves like compression.

These data have been improved in several ways. First, to focus on mechanical responses, analyses were re-done seconds after ablation. Second, to provide quantitative comparisons of junctional

© 2024. Published by The Company of Biologists under the terms of the Creative Commons Attribution License (https://creativecommons.org/licenses/by/4.0/).

responses to ablation, a PIV approach was implemented. Third, responses were found to be similar at distinct types of cell-cell junctions (straight bi-cellular junctions, curved bi-cellular junctions, and tri-cellular junctions). Fourth, suggesting a possible reason for the widespread junctional movements in response to local ablation, local ablation MT bundles that run along cell-cell junctions resulted in their rapid depolarization which coincided with movements of MT bundles of surrounding cells toward the ablation site. Please see the following text and figures:

Line 250: “To probe for compressive stress at amnioserosal AJs, we used laser ablation to cut individual AJs in DE-cad-GFP embryos and quantified responses with particle image velocimetry (PIV) analyses (Figure S5A-B and Methods). Locally cutting a cell-cell contact of the dorsal germband resulted in recoil of most surrounding junctions away from the cut site (Figure 5A-C; Movie 1), indicating tensile stress, as expected for a tissue with junctional actomyosin networks (Zulueta-Coarasa and Fernandez-Gonzalez, 2017). In contrast, locally cutting a cell-cell contact of the amnioserosa resulted in displacements of surrounding junctions toward the cut site (Figure 5A-C; Movies 2-3), indicating compressive stress.”

Line 257: “Similar responses occurred following cuts of curved bi-cellular junctions, straight bi-cellular junctions, or tri-cellular junctions (Figures S6A and 5B-C), suggesting general compression across the tissue.”

Line 264: “In embryos expressing Jupiter-GFP, a marker of MTs, local ablation at a cell-cell contact resulted in apparent MT depolymerization away from the cut site within seconds (Figure S7A, bracket; quantified in Figure S7B), and loss of MTs throughout the entirety of the ablated cells within a minute (Figure S7C). Within seconds, intact MT bundles of surrounding cells also shifted position toward the cut site (Figure S7A, white arrows). Together, these data indicate that the fully elongated amnioserosa is under compressive stress, possibly withstood by MT bundles running along cell-cell junctions.”

Line 379: “We propose that MT bundles form cortical cages around the circumferences of amnioserosal cells, and that their flexural rigidity bears compressive stress (Kikumoto et al., 2006). Pushing the sides of MT bundles against cell-cell contacts might generate curvilinear shapes in some cells, and could drive nuclei inward in others.”

Reviewer 2 Advance Summary and Potential Significance to Field:
Ray et al. examine the mechanisms that underlie the common orientation of amnioserosal (AS) cells in the Drosophila embryo. They hypothesize that compressive forces from the surrounding germband tissue, a low adhesion regime in the AS cells, and microtubule reinforcement at the boundaries of AS cell help this tissue align cells through a nematic process along the AP axis. The work is interesting and mostly well-done. A few major and minor comments are below:

We thank the reviewer for this summary and for their helpful comments below.

Reviewer 2 Comments for the Author:
1) The laser cuts that are performed to suggest AS compression need much better documentation. This is a major point of the study, but is currently shown by just one example of a cut in a single panel. This needs to be shown in a much better way – before and after images, cuts in multiple places along cells (the one AS image looks like the cut was done in an inflection point in cell curvature), etc. Quantitation should be performed.

As also discussed in response to Reviewer 1, these data have been improved in several ways. First, to focus on mechanical responses, analyses were re-done seconds after ablation. Second, to provide quantitative comparisons of junctional responses to ablation, a PIV approach was implemented. Third, responses were found to be similar at distinct types of cell-cell junctions (straight bi-cellular junctions, curved bi-cellular junctions, and tri-cellular junctions). Fourth, suggesting a possible reason for the widespread junctional movements in response to local ablation, local ablation MT bundles that run along cell-cell junctions resulted in their rapid depolarization which coincided with movements of MT bundles of surrounding cells toward the ablation site. Please see the following text and figures:

© 2024. Published by The Company of Biologists under the terms of the Creative Commons Attribution License (https://creativecommons.org/licenses/by/4.0/).

Line 250: “To probe for compressive stress at amnioserosal AJs, we used laser ablation to cut individual AJs in DE-cad-GFP embryos and quantified responses with particle image velocimetry (PIV) analyses (Figure S5A-B and Methods). Locally cutting a cell-cell contact of the dorsal germband resulted in recoil of most surrounding junctions away from the cut site (Figure 5A-C; Movie 1), indicating tensile stress, as expected for a tissue with junctional actomyosin networks (Zulueta-Coarasa and Fernandez-Gonzalez, 2017). In contrast, locally cutting a cell-cell contact of the amnioserosa resulted in displacements of surrounding junctions toward the cut site (Figure 5A-C; Movies 2-3), indicating compressive stress.”

Line 257: “Similar responses occurred following cuts of curved bi-cellular junctions, straight bi-cellular junctions, or tri-cellular junctions (Figures S6A and 5B-C), suggesting general compression across the tissue.”

Line 264: “In embryos expressing Jupiter-GFP, a marker of MTs, local ablation at a cell-cell contact resulted in apparent MT depolymerization away from the cut site within seconds (Figure S7A, bracket; quantified in Figure S7B), and loss of MTs throughout the entirety of the ablated cells within a minute (Figure S7C). Within seconds, intact MT bundles of surrounding cells also shifted position toward the cut site (Figure S7A, white arrows). Together, these data indicate that the fully elongated amnioserosa is under compressive stress, possibly withstood by MT bundles running along cell-cell junctions.”

Line 379: “We propose that MT bundles form cortical cages around the circumferences of amnioserosal cells, and that their flexural rigidity bears compressive stress (Kikumoto et al., 2006). Pushing the sides of MT bundles against cell-cell contacts might generate curvilinear shapes in some cells, and could drive nuclei inward in others.”

2) The Introduction is nicely written, with one exception. The authors say, “The most common topological defects associated with nematically ordered systems are comet-like +1/2 defects and threefold symmetric -1/2 defects”. There is zero definition of what this means – much greater effort needs to be done to explain this and the later quantitation. The one schematic is not particularly helpful.

We improved the explanation of the defects with the following text:

Line 110: “These topological defects are sites where alignment is lost. For example, elongated elements can become arranged in a local aster, referred to as a +1 defect. However, more energetically favourable configurations are comet-like +1/2 defects and threefold symmetric -1/2 defects, which are both signatures of nematically ordered systems (Balasubramaniam et al., 2022; Saw et al., 2018) (see schematics in Figure 6C).”

We explained the quantification of the defects as follows.

Line 296: “Compared with controls, α-catenin RNAi embryos also had significantly greater frequencies of amnioserosal +1/2 defects and -1/2 defects (Figure 6C), which were counted manually from vector field images.”

3) It is assumed that aPKC and Par6 are in the same place as Bazooka because they are punctate. This should be shown – reagents exist to do these colocalization experiments.

Our only reagents for detecting the endogenously expressed proteins are antibodies against the proteins and GFP-tagged versions of the proteins. We re-attempted detection of Baz by immuno-fluorescence in embryos expressing aPKC-GFP or Par-6-GFP. However, the puncta were lost with fixation. We did not attempt to compare aPKC-GFP (or Par-6-GFP) localization with that of over-expressed Baz-mCherry, since colocalization in this case might be due to the over-expression of Baz-mCherry.

The following sentence has been added:
Line 184: “The sensitivity of the puncta to fixation prevented colocalization tests of endogenously expressed proteins by immuno-staining.”

© 2024. Published by The Company of Biologists under the terms of the Creative Commons Attribution License (https://creativecommons.org/licenses/by/4.0/).

4) Line 240 “data not shown”. Believe this is against Development’s data policies.

An example of the response has been added.

Line 260: “Occasionally, junctions surrounding the cut site moved away, but performing the experiment in embryos co-expressing Histone-RFP and DE-cad-GFP revealed that this effect correlated with cutting of the nucleus below (Figure S6B).”

5) The authors should work on their figure layouts and do statistical testing in each circumstance where differences are reported (Fig. 2D, for example). The statistical test used should be reported in each figure legend, and appropriate parametric or non-parametric tests (depending on data type/distributions) used.

Figure layouts have been improved.

Where differences are reported, the results of statistical tests are now provided: please see Fig 2C, Fig 2D, Fig 3A, Fig 3C, Fig 4A, Fig 5C, Fig 6A-C, Fig 7D, FigS1A, Fig S2B-C, Fig S2E-F, and Fig S8B.

Because our approach to statistical testing required explanation, we provided this explanation in the Methods, and refer to the Methods after reporting p values in each figure legend.

Line 527:
“**Statistical analyses**
Statistical significance was calculated with both Student’s t tests (two-tailed, unpaired) using Excel (Microsoft) and Mann-Whitney U tests (www.socscistatistics.com/tests/mannwhitney/). The highest P- value (lowest significance) of the two tests is shown on graphs.”

Minor notes

a) Would pay good money to whoever rips out all the bracket keys in the keyboards in the lab (kidding, but kind of not. So many, so overused, so distracting).

We left our keyboards intact, but removed 11 superfluous brackets from the figures.

b) And so… Are white brackets missing in Fig. S2?

Thank you. The correction has been made (now Figure S4).

Second decision letter

MS ID#: DEVELOP/2023/202577

MS TITLE: Confinement promotes nematic alignment of spindle-shaped cells during Drosophila embryogenesis

AUTHORS: Tirthankar Ray, Damo Shi, and Tony Harris

I have now received all the referees reports on the above manuscript, and have reached a decision. The referees' comments are appended below, or you can access them online: please go to BenchPress and click on the 'Manuscripts with Decisions' queue in the Author Area.

The overall evaluation is positive and we would like to publish a revised manuscript in Development. Before proceeding further could you please address the request of clarifications from the two reviewers. I don't think it is necessary to address the 3rd point from Reviewer 1 by removing some data.

© 2024. Published by The Company of Biologists under the terms of the Creative Commons Attribution License (https://creativecommons.org/licenses/by/4.0/).

Reviewer 1

Advance summary and potential significance to field

Revised version Ray et al. Confinement promotes nematic alignment in the AS

The authors did a good job in revising their manuscript.
They clarified technical issues such as the RNAi mediated depletion in the AS as compared to epidermis.
They also present now nice quantification of the laser cuts showing recoil towards the cutting site. Less convincing are the data in support of a pseudostratified structure of the AS. They provide now apical-basal reconstructions. What I see in these images is a arrangement with all nuclei in pretty much the same AP position. In my understanding of a pseudostratified tissue, nuclei are on different positions along the apical basal axis.

What I am still missing is a clear line of arguments towards their conclusion that the confinement of the amnioserosa promotes the spindle-shaped alignment of cells. As much as I understand the argumentation of the authors, Figure 7 is central, where situations with more or less confinement at compared. High order is observed in situations with more confinement (wt and baz RNAi), whereas low order, in situations with low confinement (a-cat RNAi and fog mutant). I also accept that the epidermis compresses the AS.
I do not understand, how these data lead to the conclusions.

Firstly, the correlation with more or less confinement are based in part on situations which affect the adherens junctions. I find it problematic to assign the changed order solely on the confinement. The fog mutant appears clearer to me in this aspect, as it seems not to directly interfere with the cell shapes and cell arrangement.

Secondly, the authors try to conclude that cell shapes are determined autonomously, whereas the ordered arrangement arises non-autonomously by confinement. Again in my view, using the junction mutants/depleted embryos makes the argument difficult.

Thirdly, as I stated in my primary evalutation, there are some data included in the manuscript that do not really contribute to the conclusion. The analysis of the microtubule organization, the characterization of junctions in baz RNAi and a-cat RNAi embryos, even that these are interesting data. Having these extra data included makes it a harder reading and distracts from the central conclusion.

Comments for the author

.

Reviewer 2

Advance summary and potential significance to field

Ray et al. examine the mechanisms that underlie the common orientation of amnioserosal (AS) cells in the Drosophila embryo. They hypothesize that compressive forces from the surrounding germband tissue, a low adhesion regime in the AS cells, and microtubule reinforcement at the boundaries of AS cell help this tissue align cells through a nematic process along the AP axis. The revised manuscript is much improved, and the authors have seriously responded to the reviewer critiques. It would seem appropriate for acceptance pending the addressing of one outstanding point on statistical analysis -- am I correct in understanding that both parametric and nonparametric tests (Mann-Whitney and student t-test) are being run on all data regardless of suitability? And then simply the poorest (?) of the values is reported? Is this correct? Guess this could be one approach, but seems that the appropriate test for the appropriate data should be used.

Comments for the author

© 2024. Published by The Company of Biologists under the terms of the Creative Commons Attribution License (https://creativecommons.org/licenses/by/4.0/).

Am I correct in understanding that both parametric and nonparametric tests (Mann-Whitney and student t-test) are being run on all data regardless of suitability? And then simply the poorest (?) of the values is reported? Is this correct? Guess this could be one approach, but seems that the appropriate test for the appropriate data should be used.

Second revision

Author response to reviewers' comments

Responses to Reviewer 1
"The authors did a good job in revising their manuscript.
They clarified technical issues such as the RNAi mediated depletion in the AS as compared to epidermis.
They also present now nice quantification of the laser cuts showing recoil towards the cutting site."

Thank you. Your suggestions helped us make these important revisions.

"Less convincing are the data in support of a pseudostratified structure of the AS. They provide now apical-basal reconstructions. What I see in these images is a arrangement with all nuclei in pretty much the same AP position. In my understanding of a pseudostratified tissue, nuclei are on different positions along the apical basal axis."

We used the term “pseudostratified” in an unconventional way. To avoid confusion, we now describe the amnioserosal multicellular arrangement as “partially overlapping.”

"What I am still missing is a clear line of arguments towards their conclusion that the confinement of the amnioserosa promotes the spindle-shaped alignment of cells. As much as I understand the argumentation of the authors, Figure 7 is central, where situations with more or less confinement at compared. High order is observed in situations with more confinement (wt and baz RNAi), whereas low order, in situations with low confinement (a-cat RNAi and fog mutant). I also accept that the epidermis compresses the AS. I do not understand, how these data lead to the conclusions."

"Firstly, the correlation with more or less confinement are based in part on situations which affect the adherens junctions. I find it problematic to assign the changed order solely on the confinement. The fog mutant appears clearer to me in this aspect, as it seems not to directly interfere with the cell shapes and cell arrangement."

We agree about this concern, and have stated the concern in our rationale for the fog mutant analysis: “Although α-catenin RNAi embryos form continuous AJs in the amnioserosa (Figure 1B-C), their amnioserosal cell misalignment might arise from defective amnioserosal cell-cell interactions rather than abnormal germband positioning. To test the effect of germband positioning with a perturbation not expected to affect the amnioserosa autonomously, we analyzed folded gastrulation (fog) mutants.”

We also address the concern in the Discussion by referring to examinations of additional mutants not expected to directly affect adherens junctions: “Planar polarity mechanisms are unable to align amnioserosal cells when the tissue elongates against an immobile germband, as occurs when the germband dissociates in embryos depleted of α-catenin (Figure 1B-C) or of Armadillo/ß-catenin (Pope and Harris, 2008), and when the germband lacks positional information in bcd nos tsl triple mutants (Pope and Harris, 2008). Planar polarity mechanisms are also unable to align amnioserosal cells across abnormally broad areas of amnioserosa tissue, as occurs when the germband extends off-course in fog mutants (Figure S8B, 6B), and when amnioserosal tissue is induced around the entire embryo in dorsal mutants (Pope and Harris, 2008).”

© 2024. Published by The Company of Biologists under the terms of the Creative Commons Attribution License (https://creativecommons.org/licenses/by/4.0/).

"Secondly, the authors try to conclude that cell shapes are determined autonomously, whereas the ordered arrangement arises non-autonomously by confinement. Again in my view, using the junction mutants/depleted embryos makes the argument difficult."

There are multiple contexts in which amnioserosal cells elongate in the embryo surface plane despite a failure of germband extension: alpha-catenin RNAi embryos, beta-catenin mutant embryos, bcd nos tsl triple mutants, and dorsal mutants. In all of these cases, amnioserosal cell elongation is evident in the embryo surface plane, despite disruption of the germband, and despite depleting adherens junction components in two of the four cases. Although amnioserosal cells can elongate in these cases, they lack normal alignment with each other, providing evidence that germband extension promotes this alignment.

"Thirdly, as I stated in my primary evalutation, there are some data included in the manuscript that do not really contribute to the conclusion. The analysis of the microtubule organization, the characterization of junctions in baz RNAi and a-cat RNAi embryos, even that these are interesting data. Having these extra data included makes it a harder reading and distracts from the central conclusion."

In our view, these are important characterizations relevant to the central conclusion of the paper. We refer to these findings in rationales for experiments directly contributing to the central conclusion. We refer to these findings in our discussions of the central conclusion.

Characterizing microtubule associations with cell-cell junctions in Figure 3 allowed stronger proposals of microtubule function in amnioserosa tissue organization in the Discussion: “Moreover, laser cutting experiments indicate that its junctions experience compressive stress (Figure 5A-C), and implicate MT bundles in bearing the stress (Figure S7). We propose that MT bundles form cortical cages around the circumferences of amnioserosal cells, and that their flexural rigidity bears compressive stress (Kikumoto et al., 2006). Pushing the sides of MT bundles against cell-cell contacts might generate curvilinear shapes in some cells, and could drive nuclei inward in others.”

Characterizing junctions in alpha-catenin RNAi embryos in Figure 1 provided the basis for studying these embryos in relation to nematic order. Note this sentence in the Results: “To test the effect of germband positioning on amnioserosal cell alignment, we first compared the nematic order of DE-cad-positive cell-cell contacts in control and α-catenin RNAi embryos at stage 10-11. α-catenin RNAi embryos fail convergent extension of the germband due to its AJ fragmentation (Sheppard et al., 2023) (Figure 1B-C).” Note this sentence in the Discussion: “Planar polarity mechanisms are unable to align amnioserosal cells when the tissue elongates against an immobile germband, as occurs when the germband dissociates in embryos depleted of α-catenin (Figure 1B-C) or of Armadillo/β-catenin (Pope and Harris, 2008), and when the germband lacks positional information in bcd nos tsl triple mutants (Pope and Harris, 2008).”

Characterizing junctions in Baz RNAi embryos in Figure 2 provided the basis for studying these embryos in relation to nematic order. Note these sentences in the Results: “To further probe the basis of amnioserosal cell alignment, we tested the role of intact AJs within the amnioserosa. Specifically, we measured amnioserosal cell alignment in Baz RNAi embryos, in which the amnioserosa loses AJ continuity but remains confined by an intact, surrounding germband (Figure 2D).” Note this sentence in the Discussion: “Moreover, planar polarity mechanisms are linked to cell-cell contacts (Devenport, 2014), but AJ continuity is not needed for amnioserosal cells to retain global alignment of their microtubule networks in Baz RNAi embryos with amnioserosal AJ fragmentation and an intact surrounding germband (Figure 7A). Instead of junctional planar polarity within the amnioserosa, confinement by the surrounding germband seems key for the global alignment of spindle-shaped amnioserosal cells.”

Responses to Reviewer 2

"Ray et al. examine the mechanisms that underlie the common orientation of amnioserosal (AS) cells in the Drosophila embryo. They hypothesize that compressive forces from the surrounding germband tissue, a low adhesion regime in the AS cells, and microtubule reinforcement at the boundaries of AS cell help this tissue align cells through a nematic process along the AP axis. The revised manuscript is much improved, and the authors have seriously responded to the reviewer

© 2024. Published by The Company of Biologists under the terms of the Creative Commons Attribution License (https://creativecommons.org/licenses/by/4.0/).

critiques. It would seem appropriate for acceptance pending the addressing of one outstanding point on statistical analysis -- am I correct in understanding that both parametric and nonparametric tests (Mann-Whitney and student t-test) are being run on all data regardless of suitability? And then simply the poorest (?) of the values is reported? Is this correct? Guess this could be one approach, but seems that the appropriate test for the appropriate data should be used."

Thank you for your overall assessment of the revised manuscript. Your past comments helped us make important revisions.

We agree with your remaining concern about the approach to statistics. The distributions of data have been reviewed to justify the statistical test. Overall, the distributions were non-normal. Thus, we have revised the paper to only include Mann-Whitney U test results, and now state the test in the legends (as requested by Development) rather than in the methods alone. This adjustment did not change the p values reported because the Mann-Whitney U test typically produces larger p values than the student's T-test, and in the rare cases when it didn't, the p value was in the same range as previously reported.

While reviewing the use of statistical tests, three errors involving a switch from p<0.01 to p<0.001, or vice versa, were corrected in Figures 6 and 7 by changing the number of asterisks in the figure file. In the legend of Figure 7, "(*p<0.05; **p<0.001)" was also corrected to "(*p<0.01; **p<0.001)."

Third decision letter

MS ID#: DEVELOP/2023/202577

MS TITLE: Confinement promotes nematic alignment of spindle-shaped cells during Drosophila embryogenesis

AUTHORS: Tirthankar Ray, Damo Shi, and Tony Harris
ARTICLE TYPE: Research Article

I am happy to tell you that your manuscript has been accepted for publication in Development, pending our standard ethics checks.

© 2024. Published by The Company of Biologists under the terms of the Creative Commons Attribution License (https://creativecommons.org/licenses/by/4.0/).
